# Concerted conformational changes control metabotropic glutamate receptor activity

**DOI:** 10.1126/sciadv.adf1378

**Published:** 2023-06-02

**Authors:** Nathalie Lecat-Guillet, Robert B. Quast, Hongkang Liu, Emmanuel Bourrier, Thor C. Møller, Xavier Rovira, Stéphanie Soldevila, Laurent Lamarque, Eric Trinquet, Jianfeng Liu, Jean-Philippe Pin, Philippe Rondard, Emmanuel Margeat

**Affiliations:** ^1^ Institut de Génomique Fonctionnelle, Univ. Montpellier, CNRS, INSERM, 141 rue de la Cardonille, 34094, Montpellier Cedex 05, France.; ^2^ Centre de Biologie Structurale (CBS), Univ. Montpellier, CNRS, INSERM, Montpellier, France.; ^3^ Key Laboratory of Molecular Biophysics of MOE, International Research Center for Sensory Biology and Technology of MOST, College of Life Science and Technology, Huazhong University of Science and Technology (HUST), Wuhan, 430074, China.; ^4^ PerkinElmer Cisbio, Parc Marcel Boiteux, 30200 Codolet, France.

## Abstract

Allosteric modulators bear great potential to fine-tune neurotransmitter action. Promising targets are metabotropic glutamate (mGlu) receptors, which are associated with numerous brain diseases. Orthosteric and allosteric ligands act in synergy to control the activity of these multidomain dimeric GPCRs. Here, we analyzed the effect of such molecules on the concerted conformational changes of full-length mGlu2 at the single-molecule level. We first established FRET sensors through genetic code expansion combined with click chemistry to monitor conformational changes on live cells. We then used single-molecule FRET and show that orthosteric agonist binding leads to the stabilization of most of the glutamate binding domains in their closed state, while the reorientation of the dimer into the active state remains partial. Allosteric modulators, interacting with the transmembrane domain, are required to stabilize the fully reoriented active dimer. These results illustrate how concerted conformational changes within multidomain proteins control their activity, and how these are modulated by allosteric ligands.

## Teaser

Activation of a G-protein coupled receptor revealed by single molecule FRET between labeled unnatural amino acids.

## INTRODUCTION

l-glutamate represents the major excitatory neurotransmitter in the mammalian brain. It activates both ligand-gated channels and metabotropic glutamate (mGlu) receptors. The latter belong to the superfamily of G protein–coupled receptors (GPCR) and are composed of two subunits encoded by eight different genes (*1*). The mGlu receptor central role in regulating neuronal excitability and synaptic transmission makes them important targets for the treatment of neurological and psychiatric diseases (*2*, *3*). A major challenge in mGlu receptor drug discovery arises from the high conservation of their orthosteric ligand-binding site (*4*). This has directed efforts toward the development of small-molecule allosteric modulators that target other binding pockets and show higher subtype selectivity (*5*, *6*).

**Fig. 1.**
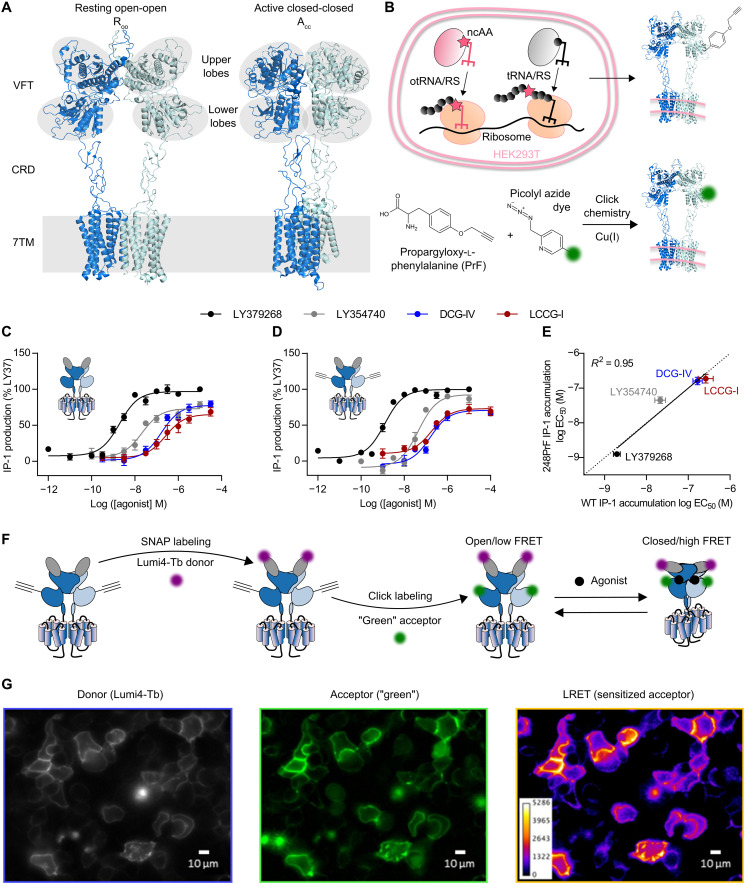
Site-specific fluorescence labeling of mGlu receptors by genetic code expansion and bioorthogonal click chemistry. (**A**) Resting open-open (R_OO_) and active closed-closed (A_CC_) structures of mGlu2 [Protein Data Bank (PDB) 7EPA and 7E9G]. The major domains are annotated including the upper and lower lobes of the VFT, the CRD, and the 7TM. The upper and lower lobes are highlighted in gray and agonist binding to the orthosteric site is shown as black spheres. (**B**) Schematic representation of genetic code expansion and bioorthogonal click chemistry. Coexpression of the orthogonal synthetase together with its cognate amber suppressor tRNA_CUA_ (otRNA/RS) in the presence of the noncanonical amino acid propargyloxy-l-phenylalanine (PrF) in HEK293T cells allows the reassignment of a premature Amber codon TAG within the ORF of the receptor gene to code for PrF. This leads to incorporation of PrF at a predefined position in the translated receptor, which subsequently reacts with a picolyl azide (pAz) dye using copper(I)-catalyzed azide-alkyne cycloaddition (CuAAC). (**C** and **D**) Ligand dose–dependent IP-1 production of SNAP-mGlu2 and SNAP-mGlu2-PrF248, respectively. IP-1 accumulation was mediated by coexpression of chimeric G_iq9_. (**E**) Correlation of log EC_50_ values obtained for SNAP-mGlu2 (C) and SNAP-mGlu2-PrF248 (D). (**F**) Schematic representation of N-terminal SNAP-labeling followed by click labeling of the lower lobe (A248) to obtain the FRET sensor used in (G). (**G**) LRET imaging of mGlu2 labeled with the Lumi4Tb-BG donor at N-terminal SNAP-tags and at position 248 in the lower lobe of the VFT with the "green" pAz acceptor at the plasma membrane of live HEK293T cells. Data in (C) and (D) represent the mean of 3 to 12 measurements ± SD.

mGlu receptors share a similar multidomain architecture and activation mechanism with other class C GPCRs activated by l–amino acids, sugars or cations such as the GPRC6A, the umami and sweet taste receptors or the calcium sensing receptor (*7*). In these receptors, orthosteric ligands bind within a cleft of the Venus flytrap domain (VFT), leading to the activation of one of the 7 transmembrane-spanning domains (7TM) via the cysteine-rich domains (CRD) (Fig. 1A). Early structural studies of isolated VFTs of mGlu receptors have shown that the upper and lower lobes of each VFT protomer come into closer proximity when bound to agonists, switching from an “open” (o) to a “closed” (c) state (Fig. 1A and fig. S1). This is accompanied by a reorientation of the two adjacent VFT protomers, increasing the distance between N termini located in the upper lobes, while the lower lobes come in closer proximity—referred to as the “resting” (R) state to “active” (A) state transition (Fig. 1A and fig. S1) (*8*–*11*). Such a model highlighting the inactive, “resting open/open” (R_OO_; Fig. 1A and fig. S1) and “active closed/closed” (A_CC_; Fig. 1A and fig. S1) conformations of the VFT dimer was recently confirmed by cryo–electron microscopy (cryo-EM) structures of nearly full-length receptors (*12*–*17*). These structures further revealed a nonconserved mechanism with regard to the reorganization of the 7TM helix bundle upon activation, compared to GPCRs from other classes (*13*, *14*).

However, fully understanding how agonists and allosteric modulators act on the initial steps of mGlu receptor activation requires taking into account receptor structural dynamics at the level of each individual domain—notably the closure of the VFT (open to closed state) in relation to the relative reorientation of the VFTs (resting to active state) but also the conformational rearrangements of the CRDs and the 7TM domains. Toward this goal, we recently used single-molecule Förster resonance energy transfer (smFRET) on receptors labeled through N-terminal SNAP-tags. We showed that the combination of an orthosteric agonist with positive allosteric modulators (PAMs; such as the small organic molecule Biphenylindanone A (BINA) or the heterotrimeric G protein) is required to maximize the residence time of the VFTs in the active orientation (*18*). This lack of stabilization of the active conformation by the orthosteric agonists alone could have several explanations. It could result from agonists promoting an intermediate conformation, where one VFT in the dimer adopts the open, while the other VFT adopts the closed state (A_OC_; fig. S1). Such A_OC_ conformations have been observed in cryo-EM structures of nearly full-length mGlu1 (*17*) and mGlu2 (*14*) as well as in crystal structures of the isolated mGlu1 extracellular domain (ECD) (*8*). Alternatively, a fraction of the dimers could remain in the resting orientation although both VFTs are closed, as observed for the agonist-bound mGlu3 extracellular domain (R_CC_; fig. S1) (*10*). The putative conformational landscape further gains complexity by the observation of an mGlu1 VFT dimer resembling an active orientation but with both VFTs being open (A_OO_; fig. S1). While these R_CC_, A_OC_, and A_OO_ conformations were observed in structural studies where putative intermediate states could be trapped, their implication in the dynamic conformational path from inactive R_OO_ to fully active A_CC_ remains to be elucidated. Several smFRET studies have made attempts to address this; however, none of these directly monitored the closure of the VFTs. The vast majority of studies used a sensor based on N-terminal SNAP-tag labeling ex cellulo in detergent micelles (*18*–*22*) or within the membrane of living cells (*23*), which undoubtedly reports on the resting to active transition but does not provide direct information on VFT closure. A few other studies further examined the conformational changes of other domains including the CRDs by smFRET (*24*, *25*) as well as the 7TMs by ensemble FRET using either fluorescent protein fusions (*26*, *27*) or organic dyes on truncated 7TMs lacking the ECDs (*28*).

To gain deeper insights into the mechanism of this initial step of mGlu2 activation, we developed FRET-based sensors to individually dissect the “open-to-closed” and “resting-to-active” transitions. Our strategy relies on the incorporation of the reactive noncanonical amino acid (ncAA) propargyloxy-l-phenylalanine (PrF) by genetic code expansion (Fig. 1B). In this way, fluorophores can be precisely conjugated to the receptor at desired positions using the bioorthogonal copper-dependent click chemistry (*24*, *29*). Screening various positions, we validated sensors allowing the detection of either the VFT closure or the VFT reorientation, by lanthanide resonance energy transfer (LRET) on the surface of living cells. By using smFRET on full-length, detergent-solubilized, fully functional receptors, we report a three-state model composed of two main conformational equilibria between the R_OO_, R_CC_, and A_CC_ states. Our data demonstrate that orthosteric agonist binding promotes the stabilization of the closed state of the VFTs to an extent correlated with their pharmacological efficacy. VFT closure allows the receptor to establish an equilibrium between the resting and active orientations. However, in the absence of G protein, a full reorientation requires the presence of the mGlu2 PAM BINA, which promotes the stabilization of the receptor in the active state. These data highlight how allosteric modulators can act on concerted conformational changes to modulate receptor activation.

## RESULTS

### Establishment of live cell-compatible click chemistry conditions for mGlu2 labeling

At first, we established conditions for the site-specific incorporation of PrF and efficient labeling by copper(I)-catalyzed azide-alkyne cycloaddition (CuAAC) using picolyl azide (pAz) dyes (Fig. 1B). We selected the ncAA PrF due to its very good stability under physiological conditions and its capability to react with aryl-azides in the presence of Cu(I) through CuAAC in a fast, highly selective, and bioorthogonal manner (*30*). We introduced single, premature amber codons (TAG) at 13 different positions within the gene of our SNAP-mGlu2 construct throughout the region coding for the VFT (fig. S2). PrF was incorporated in response to the suppression of premature TAG by cotransfection of human embryonic kidney (HEK) 293T cells with the vector coding for SNAP-mGlu2 together with a bicistronic vector coding for an engineered *Escherichia coli* tyrosyl–transfer RNA (tRNA) synthetase (PrFRS) (*31*, *32*) and three copies of the engineered *Bacillus stearothermophilus* tyrosyl-tRNA_CUA_ (*33*, *34*). By screening the various positions, we found that incorporation of PrF at seven different sites led to good incorporation yields, up to 35.9% of wild type for example for the substitution of Ala248 (fig. S2B). Furthermore, we verified the mutants’ signaling activities, using an inositol phosphate-1 (IP-1) accumulation assay reporting on a downstream cellular response mediated by chimeric G_qi9_ (*35*). IP-1 assays performed in the presence of the full agonist LY379268 revealed functional integrity of most of the tested mutants (fig. S2B). On the basis of excellent incorporation efficiency and functional integrity, we further focused our attention on the SNAP-mGlu2-PrF248 mutant to optimize the CuAAC labeling conditions. We noted that minor mGlu2 expression was detected in the absence of PrF (fig. S3A), indicating readthrough of the TAG codon, likely by tRNA_CUA_ being charged with Gln as described previously (*36*). This readthrough should be largely prevented in the presence of the engineered suppressor tRNA_CUA_, but even if being produced, this minor population would not interfere with downstream LRET and FRET analysis as no labeling of receptors lacking PrF would occur. Titrations with a set of orthosteric partial (LCCG-I, DCG-IV, and LY354740) and full agonists (glutamate and LY379268) on SNAP-mGlu2 (Fig. 1C) and SNAP-mGlu2-PrF248 (Fig. 1D) revealed similar median effective concentration (EC_50_) values based on IP-1 accumulation (Fig. 1E), demonstrating that PrF incorporation did not severely affect receptor pharmacology.

The CuAAC reaction requires the presence of Cu(I) to proceed efficiently under physiological buffer conditions (*37*, *38*). Multiple protocols have been described to reduce Cu(II) to Cu(I), stabilize it in solution and minimize its cytotoxicity as well as that of unwanted side products, e.g., reactive oxygen species. We combined two previously described strategies to minimize the required concentration of copper while maintaining satisfying reaction efficiencies. First, we synthesized pAz derivatives of the Lumi4-Tb donor together with “green” and “red” acceptors (a fluorescein and DY647 derivative, respectively; see the Supplementary Materials), as pAz increases labeling efficiencies through stabilization of a precomplex with copper (*39*). Second, we used 2-[4-{(bis[(1-tert-butyl-1H-1,2,3-triazol-4-yl)methyl]amino)methyl}-1H-1,2,3-triazol-1-yl] acetic acid (BTTAA) as a ligand to stabilize Cu(I) in solution (*40*). We found that BTTAA outperformed tris[(1-hydroxy- propyl-1H-1,2,3-triazol-4-yl)methyl]amine (THPTA) with regard to reaction efficiency (fig. S3B). Using 200 to 500 μM CuSO_4_ and 6 equivalents of BTTAA, no cytotoxicity was observed, and labeling proceeded efficiently (fig. S3, C and D). Moreover, a good labeling specificity was obtained using 4 to 6 equivalents BTTAA over CuSO_4_ compared to lower ratios (fig. S3E). Labeling with pAz-green, pAz-red, and, to a lesser extent, pAz-Lumi-Tb proceeded efficiently, as judged by fluorescence reaching a plateau and exhibiting similar intensities compared to receptors labeled at N-terminal SNAP-tags (fig. S4, A to C).

### LRET-based conformational sensors for investigating mGlu2 receptor conformational changes

Double labeling of the N-terminal SNAP-tags and PrF248 is expected to lead to a FRET signal between the upper and lower VFT lobes that will increase in the presence of agonists by bringing the two lobes into closer proximity (Fig. 1F). Using several combinations of dye pairs, a notable LRET signal was observed, specific to the presence of PrF248 and reaching saturation at only a few micromolars of pAzF dye (fig. S4, D to F). The optimal windows for calculation of the sensitized acceptor emission (fig. S4G) were determined as previously described (*41*). Using these optimized conditions, we demonstrated live HEK293T cell LRET imaging of mGlu2, labeled with 3 μM SNAP-Lumi4-Tb and 8 μM pAz-green for 25 min (Fig. 1G). We specifically chose this labeling combination as it provided a good LRET change between nonstimulated and agonist-stimulated receptors while using commercially available functionalized dyes. This confirmed the live cell compatibility of our labeling protocol, with a substantial FRET signal as a result of donor-sensitized acceptor emission between the upper and lower lobes of the VFTs.

**Fig. 2.**
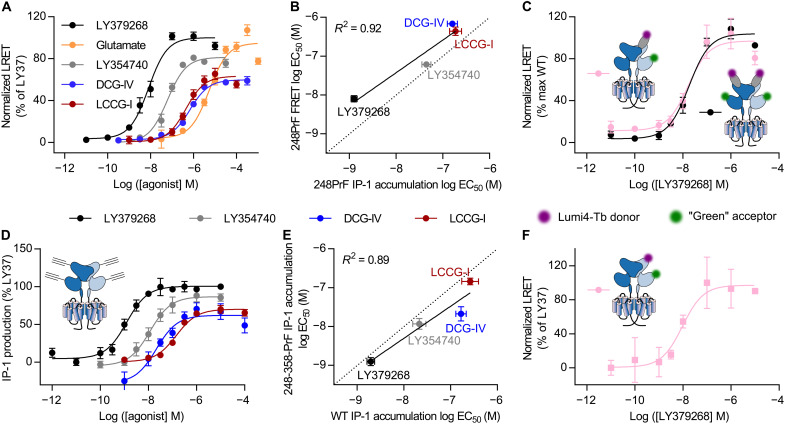
Establishment of a sensor to monitor VFT closure in a single protomer. (**A**) Ligand dose–dependent LRET changes between the upper lobes, labeled via SNAP-tags with Lumi4-Tb donor, and the lower lobes, labeled through PrF248 with pAz green acceptor. Both protomers in the dimer are labeled, giving rise to a maximum of two donor and two acceptor dyes per receptor. (**B**) Correlation of log EC_50_ values obtained from LRET reporting on VFT closure (A) and downstream signaling given by IP-1 production (see Fig. 1D). (**C**) Comparison of dose-response behavior on VFT closure measured by LRET for SNAP-mGlu2-PrF248 homodimers and specifically engineered heterodimers composed of one unlabeled (mGlu2-C2) and one labeled (SNAP-mGlu2-PrF248-C1) protomers (see the main text). (**D**) Ligand dose–dependent IP-1 production of SNAP-mGlu2-PrF248 + 358. IP-1 accumulation was mediated by coexpression of chimeric G_iq9_. (**E**) Correlation of log EC_50_ values obtained for SNAP-mGlu2-PrF248 + 358 (D) and SNAP-mGlu2 (see Fig. 1C). (**F**) Dose-dependent increase in LRET as a result of VFT closure in response to LY379268 for the engineered heterodimer, in which only one VFT protomer is labeled on PrF248 + 358. Data in (A) to (F) represent the mean of 3 to 12 measurements ± SD. WT, wild type.

Then, we performed titrations with a set of orthosteric agonists on living cells, which revealed the expected dose-dependent increase in LRET efficiency (Fig. 2A) as a result of VFT closure on the SNAP-mGlu2-PrF248 sensor. Ligand potencies (pEC_50_) obtained by LRET correlated well with the pEC_50_ obtained from a downstream cellular response monitored by IP-1 accumulation (Fig. 2B), although the latter were higher, as previously reported for the N-terminal SNAP sensor (*42*).

Together, we established a procedure for the incorporation of PrF into mGlu2 and its efficient labeling using click chemistry, compatible with living cells. Our approach allows screening for ncAA incorporation efficiency, functionality of modified receptors, and evaluation of FRET-based conformational sensors, directly on living cells.

### Monitoring mGlu2 VFT closure in a single protomer using dual ncAA labeling

Although our SNAP-mGlu2-PrF248 sensor was functional to report on agonist-induced VFT closure by LRET, it required two further improvements to become suitable for smFRET experiments, thereby exploiting the full potential of ncAA labeling to circumvent the use of the ~19.4-kDa large SNAP-tag.

For smFRET measurement, it is required to obtain dimers carrying a single donor and a single acceptor fluorophore. Therefore, we generated mGlu2 receptors with the N-terminal SNAP-tag and the PrF248 incorporated in only one protomer, while the other one remained unlabeled. For that, we took advantage of the engineered GABA_B_ quality control system we previously developed (*43*) to specifically express desired heterodimers at the cell surface. In these constructs, the C-terminal domain of the mGlu2 subunits was replaced by that of either the modified GABA_B1_ (C1) or GABA_B2_ (C2) subunits, respectively. When not heterodimerized, these two domains lead to the retention of the chimeric subunits in the endoplasmic reticulum. Therefore, only C1-C2 heterodimers can reach the cell surface. In this way, we produced heterodimeric receptors where only one protomer is labeled (SNAP-mGlu2-PrF248-C1/mGlu2-C2). We compared the changes in LRET induced by the full agonist LY379268 on VFT closure, between this construct and the symmetrically labeled construct described above (SNAP-mGlu2-PrF248 homodimer), and found similar results with regard to potency and efficiency (Fig. 2C). Such heterodimers allow therefore to monitor the VFT closure from a single FRET pair on one protomer within the functional dimer, as required for our confocal single-molecule experiments.

We then circumvented the use of the SNAP-tag for labeling by incorporating a second PrF residue within the upper lobe of the VFT. For this, we tested multiple sites within the VFTs upper lobe in combination with 248-TAG, by screening for double PrF incorporation efficiency and functional integrity of receptors (fig. S2). The SNAP-mGlu2-PrF248 + 358 mutant outcompeted other pairs with regard to yields (fig. S2) while maintaining a pharmacological profile similar to wild-type receptors (Fig. 2, D and E). After optimization of labeling conditions using 3 μM pAz-Lumi4Tb donor and 8 μM pAz-green acceptor for 25 min at 37°C (fig. S5A), we determined the optimal sensitized acceptor decay windows (fig. S5B) to perform LRET measurements. These reported on the ligand-induced closure of the VFTs in an intrasubunit fashion, as observed by an increase of LRET (Fig. 2F). This increase was expected as the distance between the Cα of residues 248 and 358 decreases from 53 to 36 Å between the R_OO_ and the A_CC_ conformations, respectively [Protein Data Bank (PDB) 7EPA for R_OO_ and 7EPB for A_CC_ conformations, respectively (fig. S6 and table S1)]. We therefore validated the capability of this sensor based on double UAA incorporation to report on the proximity between the upper and lower lobes and hereafter refer to it as the “VFT closure” sensor.

### Full agonists induce maximal VFT closure independently of allosteric modulation

While LRET is powerful to determine ligand potency and efficacy in a population of receptors, identifying and quantifying the relative population of individual conformational states require single-molecule analysis. Therefore, receptors were solubilized using a modified version of our recently published protocol (*18*) to account for the decreased expression yields due to PrF incorporation. This new strategy relies on an initial solubilization using 1% lauryl maltose neopentyl glycol (LMNG) + 0.1% cholesteryl hemisuccinate (CHS) tris followed by dilution into buffer containing glyco-diosgenin (GDN). Control LRET experiments on SNAP-tag labeled receptors confirmed that functional integrity was maintained and demonstrated that the C-terminal modifications do not impair receptor stimulation by glutamate and BINA (fig. S7, A to D). In addition, ligand-driven intersubunit VFT reorientation occurred in an identical manner on these C-terminally modified and on wild-type constructs, as shown by control smFRET experiments on SNAP-labeled receptors (fig. S8, A to E) (*18*).

**Fig. 3.**
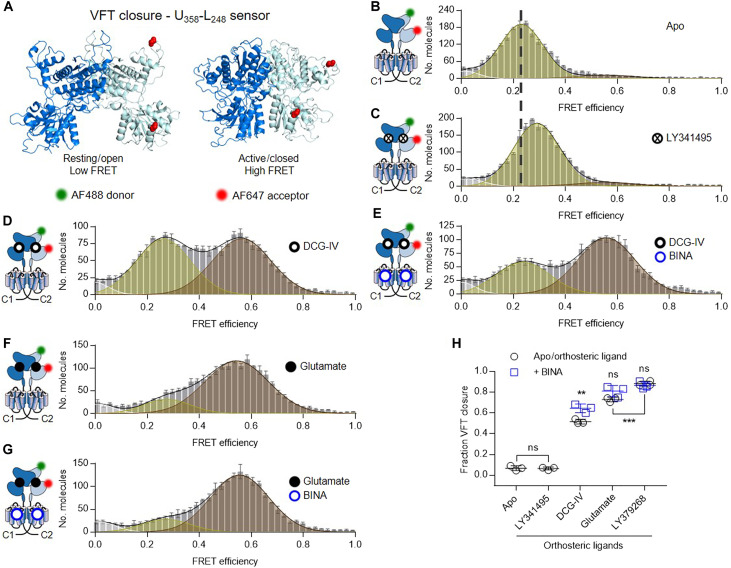
Agonists stabilize the VFT in its closed state. (**A**) Side view on structures of the VFT in the inactive resting open/open (PDB 7EPA) and active closed/closed (PDB 7E9G) conformation with residues R358 in the upper lobe and A248 in the lower lobe of one VFT replaced by PrF (red spheres). (**B** to **G**) FRET histograms of VFT closure sensor in the absence of ligand (B, Apo) and in the presence of saturating 100 μM LY341495 (C), 1 mM DCG-IV (D), 1 mM DCG-IV + 1 μM BINA (E), 10 mM glutamate (F), and 10 mM glutamate +1 μM BINA (G). FRET histograms in (B) to (G) show the accurate FRET efficiency as the mean ± SEM of three independent biological replicates each normalized to 2000 events in the DA population (*S* = 0.3 to 0.7). Histograms display the fitting with three gaussians (white = very low FRET, yellow = low FRET, brown = high FRET) together with the global fit (black). The mean FRET efficiency of the Apo condition (A) in comparison to the antagonist LY341495 (B) is indicated by a dashed line. (**H**) Measured fraction of molecules in the closed state. Shown are the means ± SD of three independent biological replicates obtained from the number of molecules found in the high FRET population (brown) over the sum of all molecules found in the low FRET (yellow) and high FRET (brown) populations. Black circles show the Apo condition and orthosteric ligands alone, while blue squares are given for agonists in the presence of 1 μM BINA. Statistical differences were determined using a one-way analysis of variance (ANOVA) with Tukey’s multiple comparisons test and are given as *P ≤ 0.01; not significant (ns) > 0.05.

We then conjugated the smFRET-compatible dyes AF488-picolyl-azide (pAz-AF488; donor) and pAz-AF647 (acceptor) in a stochastic manner to the PrF residues of the VFT closure sensor (Fig. 3A). The smFRET distribution showed a major population at a low FRET (LF) value of 0.23 in the absence of ligand, indicating that all receptors reside in an open VFT conformation in the apo state (Fig. 3B). Addition of a saturating concentration of the antagonist LY341495 led to a similar profile with a shift of the population toward a slightly higher FRET (*E* = 0.29; Fig. 3C). In contrast, the partial agonist DCG-IV led to the appearance of a second population, characterized by a high FRET (HF) state around 0.56 (Fig. 3D). In accordance with structural data (Fig. 3A), we assigned the HF and LF populations to the closed and open VFT conformations, respectively. .

Addition of the PAM BINA in the presence of DCG-IV led to an increase of the fraction of receptors appearing in the closed VFT state (Fig. 3E). In contrast, in the presence of glutamate alone, the vast majority of molecules appeared in the closed VFT state (Fig. 3F), while BINA had no significant additional effect (Fig. 3G). Similar results were obtained for LY379268 (fig. S9, A and B). Note that we excluded the possibility of significant contaminations in the observed FRET populations with undesired dimers composed of receptors being labeled on both VFTs (fig. S9, C and D). For quantitative analysis, we excluded the population at very low FRET (*E* = 0.01) (Fig. 3, B to G, and fig. S9, A and B), as it was very minor, and not sensitive to ligands (fig. S9E).

Slight differences in FRET efficiencies of the LF state were observed for the different conditions (Fig. 3, B to G, and fig. S9, A and B). This indicates that ligands led to slightly different conformations of the open state, in a way that seems uncorrelated with their relative efficiency in promoting VFT closure (fig. S9F). A small increase in FRET was found for the open state in the presence of the antagonist LY341495, indicating a slightly reduced distance between the upper and lower lobes as compared to the apo receptor. However, as expected, this ligand was unable to promote the transition of the VFT to its closed state. For all agonists tested, the closed state can be described by a single distance (*E* ~ 0.57), excluding a model where different ligands lead to different conformations of the closed state. On the contrary, our data point to a mechanism where agonist efficacies stem from their ability to push the open-closed equilibrium toward the closed state. The fraction of VFT closure, quantified as the number of molecules found in the HF population over all molecules, followed DCG-IV < glutamate < LY379268 (Fig. 3H), in accordance with measured signaling efficacies (Fig. 1C). Overall, our data point at a dynamic equilibrium between open and closed VFT conformations, where agonist action stems from their ability to stabilize the closed conformation with an efficacy related to their pharmacological action.

### Reorientation of the VFT upper lobes is favored by allosteric modulation

**Fig. 4.**
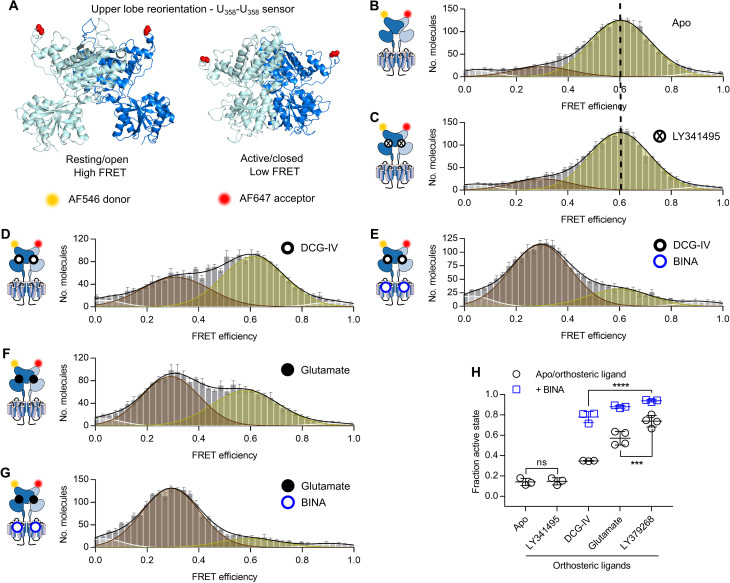
The equilibrium between resting and active upper lobe conformation follows agonist efficacy and is further promoted by allosteric modulations. (**A**) Side view on structures of the VFT in the inactive resting open/open (PDB ID 7EPA) and active closed/closed (PDB ID 7E9G) conformation with residues R358 replaced by PrF (red spheres). (**B** to **G**) FRET histograms of upper lobe sensor in the absence of ligand (B, Apo) and in the presence of saturating 100 μM LY341495 (C), 1 mM DCG-IV (D), 10 mM glutamate (E), 1 mM DCG-IV + 10 μM BINA (F), and 10 mM glutamate + 10 μM BINA (G). FRET histograms in (B) to (G) show the accurate FRET efficiency as the mean ± SEM of three independent biological replicates each normalized to 2000 events in the DA population (*S* = 0.3 to 0.7). Histograms display the fitting with 4 gaussians (white = very low FRET, yellow = low FRET, brown = high FRET, white = very high FRET) together with the global fit (black). The mean FRET efficiency of the Apo condition (A) in comparison to the antagonist LY341495 (B) is indicated by a dashed line. (**H**) Measured fraction of molecules in the fully reoriented state. Shown are the means ± SD of three independent biological replicates obtained from the number of molecules found in the high FRET population (brown) over the sum of all molecules found in the low FRET (yellow) and high FRET (brown) populations. Black circles show the Apo condition and orthosteric ligands alone, while blue squares are given for agonists in the presence of 10 μM BINA. Statistical differences were determined using a one-way ANOVA with Tukey’s multiple comparisons test and are given as ****P ≤ 0.0001, ***P ≤ 0.001, ns > 0.05.

To monitor the reorientation of the mGlu2 VFT dimer at the level of the upper lobes, we generated dimers where only Arg358 was replaced with PrF and labeled it with pAz-AF546 (donor) and pAz-AF647 (acceptor) to obtain a conformational sensor reporting on the relative orientation of the upper lobes (hereafter denoted “upper lobe” sensor) (Fig. 4A). From structural (*13*–*15*) and our previous data on receptors labeled on SNAP-tags located on the upper lobes (*18*), we expected a decrease in FRET upon receptor activation. This decrease corresponds to a distance change between the Cα of residues 358 from 66 to 78 Å between the R_OO_ (PDB 7EPA) and the A_CC_ (PDB 7EPB) conformations, respectively (fig. S6). FRET histograms showed a multimodal distribution that was best accounted for by fitting with four gaussians (Fig. 4, B to G, and fig. S10, A and B). Two minor very low and very high FRET populations (*E* = 0.03 and 0.92, respectively) were not influenced by ligands (fig. S10, C and D) and were therefore excluded from further analysis. A major HF population was found at *E*_HF_ = 0.6 in the absence of ligand and presence of the antagonist LY341495 (Fig. 4, B and C).

This HF population was partially depopulated in the presence of DCG-IV, resulting in the appearance of a second major LF population centered around *E*_LF_ = 0.3 (Fig. 4D), which was further populated in the presence of BINA (Fig. 4E). We assigned these HF and LF states to the resting and active receptor orientations, respectively. Glutamate and, to a larger extent, LY379268 had similar but more pronounced effects on the reorientation equilibrium than DCG-IV, including the allosteric effect of BINA (Fig. 4, F and G, and fig. S10, A and B). None of the ligand used had a notable effect on the recovered FRET efficiencies (*E*_HF_ and *E*_LF_). To quantify the reorientation of the VFT upper lobes, we calculated the fraction of molecules in the active population over all molecules (Fig. 4H). This confirmed a ligand-dependent impact on the equilibrium of VFT reorientation with Apo = LY341495 < DCG-IV < Glu < LY379268, both in the absence and presence of BINA.

Thus, upper lobe VFT reorientation can be described by an equilibrium between the resting and the active states. Orthosteric agonists exert differences in efficacy by shifting this equilibrium toward the active conformation, likely without stabilizing conformationally distinct states at the resolution of our two-color FRET approach. The PAM BINA that binds in the 7TM domain is strictly required to stabilize the fully reoriented VFTs in the active state, in the absence of G protein, and this effect to increase the efficacy of adopting the active orientation remains dependent on the orthosteric agonist. We note that these observations are perfectly consistent with those we previously obtained using receptors labeled on the upper lobes via N-terminal SNAP-tags (*18*).

### The lower lobe VFT reorientation limits ligand efficacy but is favored by an allosteric modulator

**Fig. 5.**
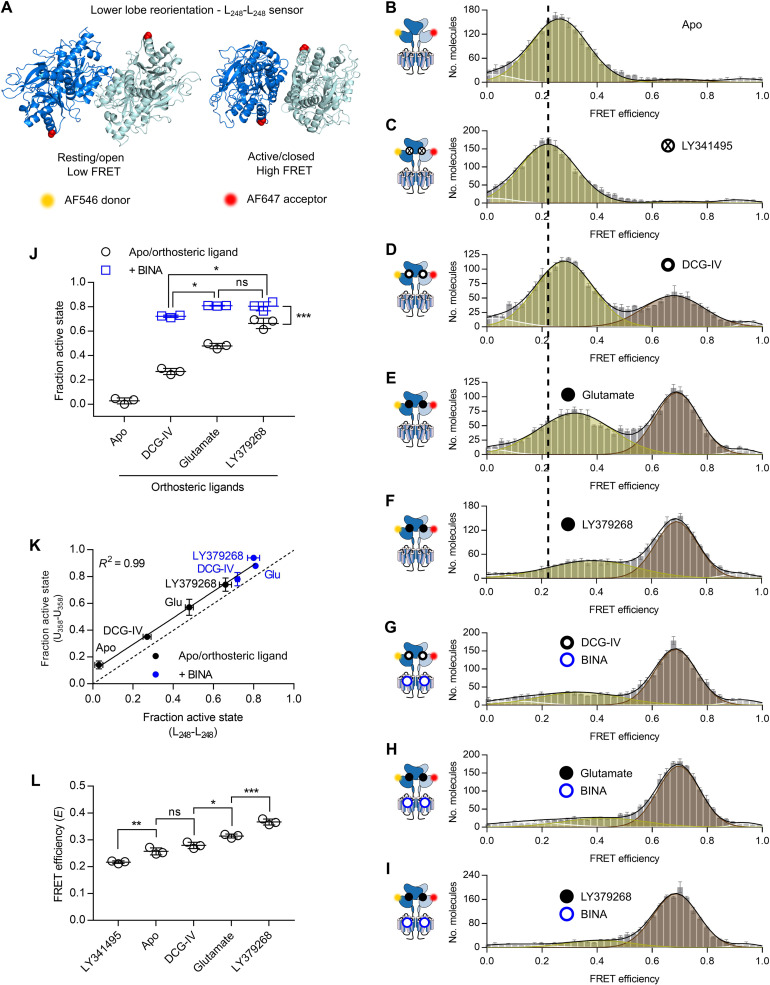
The lower lobe sensor reveals the agonist-dependent equilibrium between two distinct resting and one active state. (**A**) Bottom view on structures of the VFT in the inactive resting open/open (PDB ID 7EPA) and active closed/closed (PDB ID 7E9G) conformations with residues A248 replaced by PrF (red spheres). (**B** to **I**) FRET histograms of lower lobe sensor in the absence of ligand (Apo, B) and in the presence of saturating 100 μM LY341495 (C), 1 mM DCG-IV (D), 10 mM glutamate (E), 100 μM LY379268 (F), 1 mM DCG-IV + 10 μM BINA (G), 10 mM glutamate +10 μM BINA (H), and 100 μM LY379268 + 10 μM BINA (I). FRET histograms show the accurate FRET efficiency (mean ± SEM) of three independent biological replicates, normalized to 2000 events in the DA population (*S* = 0.3 to 0.7). Histograms display the fitting with four gaussians (white = very low FRET, yellow = low FRET, brown = high FRET, white = very high FRET) together with the global fit (black). (**J**) Measured fraction of molecules in the fully reoriented/active state, as the means ± SD of three independent biological replicates obtained from the number of molecules found in the high FRET population (brown) over the sum of all molecules. Black circles, the Apo condition and orthosteric ligands alone; blue squares, agonists in the presence of 10 μM BINA. Statistical differences were determined using a one-way ANOVA with Tukey’s multiple comparisons test, given as ***P ≤ 0.001, **P ≤ 0.01, *P ≤ 0.05, ns > 0.05. (**K**) Correlation of the fraction of molecules in the active state obtained from the upper lobe sensor (see Fig. 4H) and the lower lobe sensor (**J**). (**L**) Mean FRET efficiency of the low FRET population at different ligand conditions [yellow gaussian in (B) to (F)].

Next, we created a VFT intersubunit sensor reporting on the reorientation of the lower lobes by installing AF546 (donor) and AF647 (acceptor) fluorophores at Ala248 after incorporation of PrF (hereafter denoted “lower lobe” sensor) (Fig. 5A). Here, we predicted a shift from low to high FRET upon receptor activation, reflecting the reorientation of the VFT dimer from the resting to the active orientation. This change is characterized by a distance reduction between the Cα of residues 248 from 75 to 48 Å between the R_OO_ (PDB 7EPA) and the A_CC_ (PDB 7EPB) conformations, respectively (fig. S6).

Accordingly, a major population centered at a low FRET value of *E*_LF_ = 0.26 was obtained for the apo receptor (Fig. 5B). Again, two minor very low and very high FRET populations (*E* = 0.03 and 0.92, respectively; fig. S11, A and B) were observed, but as they were not influenced by ligands, they were excluded from further analysis. A saturating concentration of LY341495 slightly but significantly reduced this mean FRET efficiency of the main population to 0.22, indicating a further separation of the lower lobes (Fig. 5C). Agonists led to the appearance of a HF population centered around *E*_HF_ = 0.69, a value that remained constant for different agonist conditions, pointing at a single distance between the lower lobes in the active, reoriented state (Fig. 5, D to I). We again calculated the fraction of VFT reorientation as the number of molecules in the active population over the sum of all molecules (Fig. 5J). This quantification confirmed the ligand-dependent impact on the equilibrium of VFT reorientation observed earlier for the upper lobe sensor, with a remarkable correlation between the values recovered between these two conformational sensors (Fig. 5K). This included the allosteric effect observed in the presence of BINA, which stabilizes the fully reoriented VFTs in the active state, an effect that depends on the orthosteric agonist, as BINA has no effect on reorientation by itself (fig. S11C).

For all orthosteric ligands, the LF population remained significantly populated, but its FRET value (*E*_LF_), reporting on the distance between the lower lobes, varied significantly between 0.22 and 0.36 (Fig. 5, B to F) and correlated with the efficacy of the ligands (Fig. 5L).

We propose that this shift in *E*_LF_ stems from the transition of the receptor from a R_OO_ to a R_CC_ state, for which the distance between the labels on the lower lobes would be reduced by a few angstroms, in accordance with VFT crystal structures obtained on mGlu3 (fig. S6, compare R_OO_ and R_CC_ for lower lobe sensor) (*10*). This hypothesis is further supported by the observation that in the presence of agonists, the fraction of VFTs in the closed state (VFT closure sensor; Fig. 3F) is higher than that of VFTs in the active state (upper or lower lobes sensors, Figs. 4F and 5J, respectively). This implies that some of the VFTs are simultaneously closed and in the resting orientation (R_CC_). In the presence of antagonist, the R_OO_ conformation is preferentially populated (with *E*_LF_ = 0.22; Fig. 5C), while with LY379268, the fraction of receptors remaining in the resting state is found in the R_CC_ conformation (with *E*_LF_ = 0.36; Fig. 5F).

These data confirm a model where agonist binding leads to a stabilization of the closed state of the VFT with different efficacies (Apo < DCG-IV < Glu < LY379268), which allows the receptor to establish an equilibrium between the R_CC_ and the A_CC_ states. Addition of the PAM BINA stabilizes the A_CC_ state, by pushing the R_CC_ ↔ A_CC_ equilibrium. As a consequence, the law of mass action leads to a decrease in the R_OO_ population, to maintain the R_OO_ ↔ R_CC_ equilibrium. This effect is observed in the presence of the partial agonist DCG-IV, for which the addition of BINA, which leads to the expected resting to active transition (Figs. 4H and 5J), is accompanied by a closure of the VFT (Fig. 3H).

## DISCUSSION

Here, we presented a live cell-compatible click chemistry approach to site-specifically label mGlu receptor protomers with donor and acceptor fluorophores. This approach allowed us to screen for suitable positions to establish FRET sensors, directly reporting on conformational rearrangements of individual receptor domains by LRET and smFRET. With this approach, we analyzed two important conformational changes known to be associated with mGlu receptor activation: (i) the closure of the VFT domains and (ii) their relative movement to reach the active state. We show that full agonists strongly favor the adoption of a closed VFT, but the fraction of receptors visiting the active orientation remains limited in the absence of G protein. Only in the presence of the PAM BINA bound to the 7TM domain can most of the agonist-occupied dimers be found in the fully reoriented active conformation. These data illustrate how PAMs modulate agonist potency and efficacy, with regard to the resting to active conformational transition, by controlling the concerted conformational changes between closure of the VFTs and their reorientation.

Our study allowed us to validate mGlu2 LRET biosensors suitable for monitoring the VFT closure of receptors at the surface of live cells. Measuring such a precise conformational change induced by orthosteric ligand binding on the mGlu receptor was previously inaccessible. The previous LRET biosensors mainly relied on the use of large SNAP-tags at the N-terminal end of the protomers, reporting solely on the upper lobe reorientation of the VFT dimer in an intersubunit fashion upon ligand binding (*41*, *42*). We achieved site-specific and bioorthogonal labeling of the VFT using an optimized CuAAC protocol with cell impermeable dyes. The CuAAC remains one of the fastest and most specific reactions (*44*), and our optimized protocol allowed us to limit the use of copper to prevent cellular toxicity (*45*). This approach opens up new possibilities to study ligand pharmacology by directly looking at receptor activation at the level of individual domain rearrangements, which can then be related to downstream cellular responses. These sensors allow discrimination between agonists of different efficacies and reveal the activity of PAMs. Our LRET biosensors are further compatible with high-throughput screening for drug discovery.

**Fig. 6.**
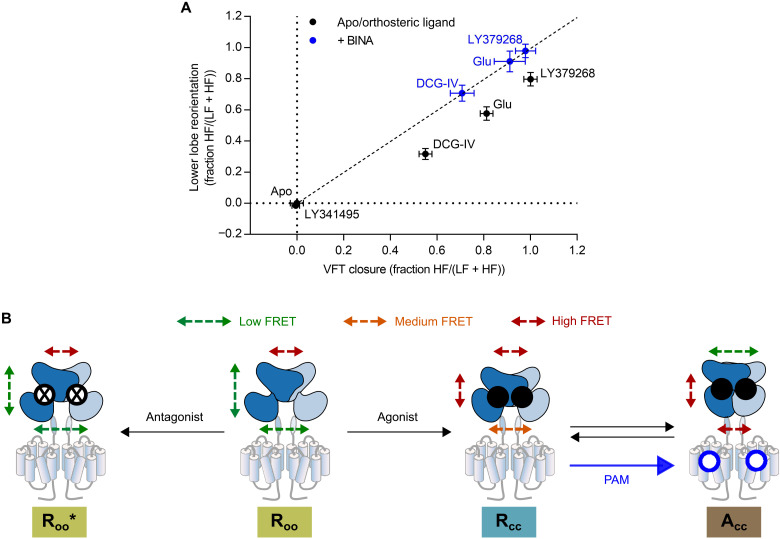
Three-state model of mGlu2 activation. (**A**) Correlation of VFT closure and lower lobe reorientation illustrates that agonists binding leads to an equilibrium between open and closed VFT, with full agonists favoring the closed state (black circles). In contrast, a maximal stabilization of the reoriented state requires the synergistic action of the PAM (BINA, blue circles). Shown are the normalized means (VFT closure: min = apo, max = LY379268; reorientation: min = apo, max = LY379268 + BINA) ± SD of three independent biological replicates. (**B**) The initial step of mGlu2 activation can be described by a three-state equilibrium between the R_oo_ (resting/open-open), the R_cc_ (resting/closed-closed) and the A_cc_ (active/closed-closed) states. Agonists activate the receptor through establishment of the R_oo_ to R_cc_ equilibrium, where agonists populated the R_cc_ state in a manner reflecting their pharmacological efficacies. In the absence of allosteric modulators, the VFTs then readily visit the A_cc_ state. PAMs exclusively act on the R_cc_ to A_cc_ equilibrium by increasing the residence time of molecules in the A_cc_ state. Antagonists induce a slight closure of the VFTs together with a small increase in separation of the lower lobes (R_oo_*). The arrows on the cartoons indicate the degree of FRET. The boxes indicating the different states are colored according to the respective FRET states shown in Figs. 3 to 5.

In our study, we focused on the initial step of mGlu2 activation, e.g., the major intra- and intersubunit rearrangements occurring within the VFT dimer of this class C GPCR. Through the analysis of these conformational rearrangements on single receptors solubilized in proper detergents, we show that mGlu2 VFT activation is governed by two conformational equilibria between three different states. Overall, our data point to a model where orthosteric and allosteric ligands act differently on the equilibria between these three main states, corresponding to the R_OO_, R_CC_, and A_CC_ conformations (Fig. 6). Orthosteric ligands appear to primarily act on the open/closed equilibrium (R_OO_ ↔ R_CC_). They promote and stabilize the closure of the VFT domains to an extent that follows their pharmacological efficacy (Figs. 1C and 6A). The three tested agonists (DCG-IV, glutamate, and LY379268) led to a high degree of VFT closure (52, 73, and 88% for the VFT closure sensor, respectively; Fig. 3H), while their ability to reorient the receptor to the active state is more limited (35, 57, and 74% for upper lobe sensor, respectively; Fig. 4H). The PAM BINA acts on a second equilibrium, between the R_CC_ and A_CC_ conformations (Fig. 6B). In its presence, the R_CC_ state is destabilized, the receptor reorientation toward its active A_CC_ state is favored, and therefore the closure of the VFT by agonists is more directly linked to the reorientation. Accordingly, in the presence of the PAM, a direct correlation appears (Fig. 6A) between the extent of VFT closure (measured by the VFT closure sensor; Fig. 3H) and dimer reorientation (measured by the upper lobe sensor; Fig. 4H).

The variations in the mean FRET efficiency of the LF state on our VFT closure sensor (Fig. 3) indicate the adoption of distinct open conformations for the different orthosteric ligands tested, which do not correlate with agonist efficacies (fig. S9F). We further identified an additional antagonist-bound open state (R_oo_* in Fig. 6B), which is characterized by a slight decrease in the distance between upper and lower lobes indicating a partial VFT closure (Fig. 3C) and an increase in the lower lobe separation (Fig. 5C). These differences correlate well with the two recently described inactive mGlu2 structures, where the distance between the Cα atoms of our VFT closure sensor shows a 1.3 Å decrease and the lower lobe distance increases by 7.1 Å, when bound to antagonist (*14*) as compared to the ligand-free VFT (fig. S12) (*15*). In contrast, the closed HF state is characterized by a single mean donor/acceptor distance and thus likely represents a highly similar single conformation no matter what agonist is bound.

Notably, our data do not provide evidence for receptors visiting the closed VFT state in the absence of ligand and presence of antagonist (Fig. 3, B and C). This points to a ligand-induced establishment of a conformational equilibrium rather than agonists stabilizing a preexisting closed state, although one cannot rule out that our approach may have missed a very small fraction of the latter. This further underlines the idea that the VFTs constantly oscillate between distinct open and a single fully closed state when bound to orthosteric ligands. Such a proposal is consistent with the observation that mGlu constitutive activity, as observed with mGlu5 and mGlu1, is not inhibited by orthosteric antagonists and thus does not result from a spontaneous closure of the VFTs but rather from a direct activity of the 7TM domain (*46*–*49*).

We showed that BINA or G protein alone have no effect on the resting to active transition of the VFT domains [fig. S11C and (*18*)]. Thus, both BINA and G protein act as allosteric modulators increasing agonist potency, and its efficacy to stabilize the VFTs reoriented and fully active state (*18*). The effect of the PAM BINA can therefore be explained by priming the 7TMs for engaging with G protein, a process that under native conditions in the absence of such a PAM is accomplished through the combined action of glutamate on the VFTs and G protein on the 7TMs. The fact that we find full agonists only acting partial on VFT reorientation is likely a result of the absence of G protein and thus a lack of its allosteric effect on the VFT conformation. Accordingly, this effect is recovered by the presence of BINA. This provides important information on the role of allostery in mGlu receptor signaling and the pharmacological effect of such PAMs. Such PAMs could act by lowering the effective threshold of glutamate required to activate the receptor, by enhancing the kinetics to reach its active, signaling competent state and/or by increasing the time receptors spend in this state. Nevertheless, more detailed information on the kinetics and spatial organization of G protein activation by individual receptors is required to explain the physiological relevance of this allostery and provide rational pharmacological routes to control receptor activity in the brain.

Overall, our data demonstrate that the combination of genetic code expansion and click chemistry allows establishing FRET sensors to gain structural information with precision of a few angstroms and facilitates mapping of the conformational landscape of GPCRs under various different conditions using smFRET. In addition, it provides access to the underlying conformational dynamics of different states, their interconnection, and how they are individually influenced by orthosteric and allosteric modulators. This enables to complement high-resolution structural information obtained on stabilized receptors to dissect their mechanism of activation.

## MATERIALS AND METHODS

### Materials

Aminoguanidine hydrochloride, copper(II) sulfate (CuSO_4_), (+)-sodium l-ascorbate, tris[(1-benzyl-1*H*-1,2,3-triazol-4-yl)methyl]amine, THPTA, l-glutamate, and chemicals used for synthesis were purchased from Sigma-Aldrich (St. Louis, MO, USA) unless stated otherwise. LY379268, LY354740, DCG-IV, L-CCG-I, and BINA were purchased from Tocris Bioscience (Bristol, UK). O^6^-benzylguanine and amine derivatives of Lumi4-Tb, green (fluorescein derivative), red (DY647 derivative) fluorophores, as well as Tag-lite buffer were from PerkinElmer (Codolet, France). 4-PrF was initially synthesized according to (*31*) and later purchased from Iris Biotech GmbH (Marktredwitz, Germany). BTTAA was synthesized as described in (*40*). pAz-Lumi4-Tb, pAz-green, and pAz-red were synthesized as described in the Supplementary Protocol. pAz-AF488, pAz-AF546, and pAz-AF647 were purchased from Jena Bioscience (Jena, Germany). LMNG and CHS tris salt were purchased from Anatrace (through CliniSciences, France). GDN was purchased from Avanti Polar Lipids through Merck.

### Plasmids

The pcDNA plasmid encoding human mGlu2 with N-terminal FLAG (Sigma-Aldrich) and SNAP-tags (New England Biolabs) was a gift from PerkinElmer (Codolet, France). The pRK5 plasmid encoding the rat SNAP-mGlu2 and the construction of the rat SNAP-mGluR2-C1KKXX and SNAP-mGluR2-C2KKXX plasmids were described in (*50*). Premature Amber mutations were introduced using the QuikChange Multi site-directed mutagenesis kit from Agilent Technologies (Santa Clara, CA, USA) according to the manufacturer’s protocol. The bicistronic pcDNA vector coding for the engineered *E. coli* tyrosyl-tRNA synthetase [PrFRS, pPR-EcRS2 according to (*31*), and including the enhancing mutation R265 according to (*32*)] and three copies of the engineered *B. stearothermophilus* tyrosyl-tRNA_CUA_ (*33*) was generated starting from the corresponding pET21 plasmid provided by P. Paoletti’s lab (IBENS, Ecole Normale Supérieure, Paris, France), which was designed in T. P. Sakmar’s lab (Rockefeller University, New York, USA) (*34*). The generation of the pRK5 plasmid coding for the high-affinity glutamate transporter EAACI was described in (*51*). Samples for smFRET experiments were prepared using the pIRE4-PGK-EPrFRS provided by I. Coin [obtained by mutagenesis of the aminoacyl-tRNA synthetase to Thr^37^, Ala^183^, and Leu^186^ in the pIRE4-Azi plasmid described in (*52*)].

### Cell culture, transfection, and incorporation of UAA

Adherent HEK293T cells (American Type Culture Collection CRL-3216, LGC Standards S.a.r.l., France) were cultured in Dulbecco’s modified Eagle’s medium (DMEM; Thermo Fisher Scientific) supplemented with 10% fetal bovine serum (FBS; Sigma-Aldrich) at 37°C with 5% CO_2_. HEK293T cells were transfected using Lipofectamine LTX with Plus Reagent (Thermo Fisher Scientific) in 96-well F-bottom black plates (Greiner Bio-One). A total of 45,000 cells were seeded in 100 μl of DMEM per well 24 hours before transfection. Transfections were performed using 255 ng of total DNA per well with a 10:6:1 ratio of vectors coding for mGluR2:PrFRS-tRNA_CUA_:EAACI in 30 μl of Opti-MEM I reduced serum medium (Thermo Fisher Scientific). A total of 0.255 μl of Plus Reagent per well were added and incubated for 10 min at room temperature (RT) in the dark. Then, 0.75 μl of Lipofectamine LTX per well was added, followed by an additional incubation for 30 min at RT in the dark. Last, the DNA-lipid complexes were added to the cells and incubated at 37°C for 6 hours. Then, the medium was replaced with 100 μl fresh DMEM supplemented with 0.5 mM PrF. The medium was further exchanged with fresh PrF-containing medium after 24 and 48 hours.

For tr-FRET microscopy, 100,000 cells per well were seeded in Nunc Lab-Tek chambered coverglass eight-well plates (Thermo Fisher Scientific) 24 hours before transfection. Transfection and expression were carried out as described above but using 80 μl of total transfection mixture, which were added to 200 μl of DMEM per well. Seventy-two hours after transfection, medium was exchanged to DMEM supplemented with GlutaMAX (Thermo Fisher Scientific) without PrF and incubated for 2 hours at 37°C and 5% CO_2_ before labeling.

For smFRET measurements, 500,000 cells per well were seeded in 2 ml of Gibco DMEM, high glucose, GlutaMAX supplement, pyruvate (Thermo Fisher Scientific, France), and 10% (v/v) FBS (Sigma-Aldrich, France) on six-well plates 24 hours before transfection. Transfections were carried out using a total of 2 μg per well at a 1:1 ratio of pRK5-rat-mGlu2:pIRE4-PGK-EPrFRS DNA, diluted in 200 μl of JetPrime Buffer together with 4 μl of JetPrime transfection reagent (Polyplus-transfection SA, Illkirch-Graffenstaden, France). The transfection mixture was incubated at RT for 15 min before addition to the cells. After 6 and 24 hours, the medium was exchanged to fresh DMEM GlutaMAX + 10% FBS containing 0.5 mM of PrF, and expression continued until 48 hours after transfection. Expression of C-terminally modified rat mGluR2-248TAG-358TAG-C1KKXX and mGluR2-C2KKXX constructs together with pIRE4-PGK-EPrFRS was performed for 72 hours with medium exchange after 6, 24, and 48 hours at a 4:1:5 DNA ratio, respectively. Two hours before labeling, medium was exchanged to Gibco DMEM, high glucose without phenol red, supplemented with GlutaMAX and pyruvate (Thermo Fisher Scientific, France), and incubated at 37°C and 5% CO_2_. Expression of receptors labeled via the N-terminal SNAP-tags for functional characterization after solubilization and smFRET measurements was carried out as described above but using 4 μl of Lipofectamine 2000 (Thermo Fisher Scientific, France) per 2 μg of total pRK5-rat-SNAP-mGlu2 in a total of 200 μl of OptiMEM medium for transfection. Expression was achieved in complete medium without PrF for a total of 48 hours.

### IP measurements and cytotoxicity assay

IP-1 accumulation was measured using the IP-One HTRF assay kit (#62IPAPEC, PerkinElmer, Codolet, France), following the official protocol from the manufacturer after cotransfection of indicated plasmids with G_qi9_ as previously described (*53*). Cytotoxicity was determined by staining with propidium iodide (2 μg/ml) together with Hoechst 33342 (5 μg/ml) for 15 min followed by a single wash with phosphate-buffered saline. Detection of the fluorescence was done using an Infinite F500 spectrofluorometer (Tecan).

### SNAP-tag labeling for quantification and LRET measurements

Labeling with a single fluorophore was performed on adherent cells at 37°C for 1 hour using the indicated concentrations of SNAP-Lumi4-Tb, SNAP-green, or SNAP-red diluted in Tag-lite buffer. Double labeling was performed using 100 nM SNAP-Lumi4-Tb and 60 nM SNAP-green. After labeling, cells were washed three times with Tag-lite buffer.

### Labeling of PrF-containing receptors with pAz dyes for LRET

The final CuAAC labeling mixture was composed of 1.5 mM aminoguanidine hydrochloride, 2 mM BTTAA, and 0.36 mM CuSO_4_ together with 2 mM (+)-sodium L-ascorbate in Tag-lite buffer. Single labeling was achieved by subjecting cells to the labeling mixture containing either 3 μM pAz Lumi4-Tb, pAz-green, and pAz-red. For double labeling, 3 μM Lumi4-Tb donor and 8 to 10 μM either pAz-green or pAz-red acceptor were used. Labeling was carried out for 25 min at 37°C in the dark. Cells were subsequently washed three times with Tag-lite buffer before addition of ligands. For double labeling through the SNAP-tag and PrF, cells were first labeled via the SNAP-tag using 300 nM SNAP-Lumi4-Tb or 100 nM SNAP-green, washed once with Tag-lite buffer, and then labeled via CuAAC as described above, followed by three washes before adding ligands.

### Ensemble fluorescence and LRET measurements

An Infinite F500 spectrofluorometer (Tecan) was used to measure the emission of Lumi4-Tb at 620 nm with a 50-μs delay after excitation at 340 nm using an integration time of 400 μs, green at 520 nm after excitation at 485 nm using an integration time of 1000 μs, and red at 665 nm after excitation at 610 nm using an integration time of 1000 μs. Sensitized acceptor emission from LRET between Lumi4-Tb and green was measured at 520 nm with a 50-μs delay after excitation at 340 nm using an integration time of 400 μs and between Lumi4-Tb and red at 665 nm with a 50-μs delay after excitation at 340 nm using an integration time of 400 μs (*50*).

LRET measurements of PrF-containing sensors and the SNAP sensor after receptor solubilization were performed on a PHERAstar FS microplate reader (BMG LABTECH) as described (*18*, *41*, *42*). After Lumi4-Tb donor excitation with a laser at 337 nm, the donor emission was collected at 620 nm and the sensitized acceptor emission at 520 nm for green or 665 nm for red in 5-μs intervals for a total of 2.5 s. LRET was calculated as the ratio of the sensitized acceptor emission integrated from 50 to 100 μs over 1200 to 1600 μs after donor excitation for the pure SNAP sensor, from 50 to 100 μs over 300 to 500 μs for SNAP with PrF, and from 50 to 75 μs over 330 to 550 μs for the PrF248 + 358 sensor.

### LRET microscopy

The microscope used to image LRET was described previously (*54*). Briefly, green was excited at 470 to 40 nm using a 495LP nm dichroic filter and imaged through a 520- to 15-nm filter, while “Lumi4-Tb” was excited at 350 to 50 nm using a 400LP dichroic filter and imaged through a 550- to 32-nm filter. For LRET Lumi4-Tb was excited at 350 to 50 nm using a 400LP nm dichroic filter, and the sensitized acceptor emission was collected using a 520 to15 nm.

### Labeling, preparation of membrane fractions, and receptor solubilization

Before solubilization from crude membranes, receptors were labeled for 1 hour at 37°C with either 100 nM SNAP-Lumi4-Tb and 60 nM SNAP-green in Gibco DMEM, high glucose, GlutaMAX supplement, and pyruvate (Thermo Fisher Scientific, France) for LRET measurements or 600 nM BG-Cy3B and 300 nM SNAP-red in Gibco DMEM, high glucose without phenol red, supplemented with GlutaMAX, and pyruvate (Thermo Fisher Scientific, France) for SNAP-sensor smFRET measurements. Labeling of incorporated PrF for smFRET measurements was achieved as described above using 2.5 μM pAz-AF488 and 7.5 μM pAz-AF546 (PrF358 upper lobe sensor and PrF248 lower lobe sensor) or 2.8 μM pAz-AF488 and 7.2 μM pAz-AF647 (PrF248 + PrF358 VFT closure sensor) in acquisition buffer [20 mM Tris-HCl (pH 7.4), 118 mM NaCl, 1.2 mM KH_2_PO_4_, 1.2 mM MgSO_4_, 4.7 mM KCl, and 1.8 mM CaCl_2_]. After labeling, adherent cells were washed three times with Dulbecco’s phosphate-buffered saline (DPBS) without Ca^2+^ and Mg^2+^ (Thermo Fisher Scientific, France) for 5 min at RT each, followed by detachment of the cells in DPBS using a cell scraper. Cells were then collected by a 5-min centrifugation at 1000*g* at RT, resuspended in cold lysis buffer [10 mM Hepes (pH 7.4) and cOmplete protease inhibitor] on ice, incubated for 30 min, and frozen at −80°C. The following day, the lysis mixture was thawed in the fridge during 1 hour, followed by 30 passages through a 200-μl pipette tip on ice. After two rounds of centrifugation at 500*g*, 4°C for 5 min each, the supernatant was centrifuged for 30 min, 4°C at 21,000*g* to collect the crude membranes. These were then overlaid once with acquisition buffer, recentrifuged for 5 min, and flash frozen in liquid nitrogen after removal of the supernatant. Fractions were stored at −80°C until solubilization of receptors.

Receptors were solubilized using 10 μl of acquisition buffer containing 1% LMNG (w/v) and 0.1% CHS tris (w/v) per membrane fraction (corresponding to cells cultured in one well of a six-well plate) for 15 min on ice. Subsequently, the solubilization mixture was centrifuged for 10 min at 4°C and 21,000*g*, and the supernatant was mixed with 90 μl of acquisition buffer containing 0.11% GDN (w/v). The diluted sample was then passed through a Zeba Spin Desalting Column (7-kDa cutoff; Thermo Fisher Scientific, France) equilibrated in acquisition buffer containing 0.005% LMNG (w/v), 0.0005% CHS tris (w/v), and 0.005% GDN (w/v). The eluate was then diluted 10 times in acquisition buffer and stored on ice in the dark.

### Functional integrity of solubilized receptors by LRET

The functional integrity of solubilized receptors was verified by LRET measurements after SNAP-tag labeling with SNAP-Lumi4-Tb and SNAP-green as described above. Briefly, solubilized wild-type rat receptors and C1/C2 heterodimers were further diluted two and four times in acquisition buffer together with increasing concentrations of glutamate or glutamate +10 μM BINA on white 384-well plates (polystyrene, flat-bottom, small volume, medium-binding, Greiner Bio-One SAS, France). Dose-response curves at different time points after storage of the samples at RT were obtained by LRET acquisitions on a Spark 20M (Tecan) through calculation of the ratio of the integrated sensitized acceptor emission at 535/25 nm at 100 to 500 μs over 1200 to 1600 μs after donor excitation at 340/20 nm.

### PIE-MFD smFRET setup

smFRET experiments with pulsed interleaved excitation (PIE)–multiparameter fluorescence detection (MFD) were performed on a homebuilt confocal microscope (*55*) using the SPCM 9.85 software (B&H) as described previously (*18*). Modifications are described in the following. A combination of 530/20 (530AF20, Omega Optical, Brattleboro, VT, USA) and 530/10 nm (FLH532-10, Thorlabs, Maisons-Laffitte, France) bandpass filters was used for Cy3B and AF546 excitation. A 488/10 (Z488/10 X, Chroma, Bellows Falls, VT, USA) bandpass filters was used for AF488 excitation. A 635/10 (FLH635-10, Thorlabs, Maisons-Laffitte, France) bandpass filter was used for red and AF647 excitation. The excitation power was 30 μW (prompt at 535 nm) and 12 μW (delayed at 635 nm) for Cy3B /red; 50 μM (prompt at 535 nm) and 15 μW (delayed at 635 nm) for AF546/AF647; 25 μW (prompt at 488 nm) and 12 μW (delayed at 635 nm) for AF488/AF647 at the entrance into the microscope. Inside the microscope, the light was reflected by dichroic mirrors that match the excitation/emission wavelengths of the respective fluorophore combinations (Cy3B/AF546 with red/AF647: FF545/650- Di01, Semrock, Rochester, NY, USA and AF488 with AF647: FF500/646-Di01, Semrock, Rochester, NY, USA) and coupled into a 100×, numerical aperture 1.4 objective (Nikon, France). The following emission filters were used: Cy3B/AF546 parallel ET BP 585/65, perpendicular HQ 590/75 M (Chroma, Bellows Falls, VT, USA); AF488 parallel 535/50 BrightLine HC, perpendicular 530/43 BrightLine HC (Semrock, Rochester, NY, USA); red/AF647 parallel and perpendicular FF01-698/70-25 (Semrock, Rochester, NY, USA). Dual color emission was separated using FF649LP long pass filters (parallel and perpendicular, Semrock, Rochester, NY, USA) for Cy3B/AF546 with red/AF647 and AT608LP (parallel, Chroma, Bellows Falls, VT, USA) together with FF560LP (perpendicular, Semrock, Rochester, NY, USA).

### smFRET measurements

smFRET measurements were performed on SensoPlate 384-well plates (nontreated, Greiner Bio-One, France) passivated with bovine serum albumin (1 mg/ml) in acquisition buffer with 0.0025% LMNG (w/v), 0.00025% CHS tris (w/v), and 0.0025% GDN (w/v) for at least 1 hour before sampling application. Samples containing 30 to 100 pM labeled receptors were measured in acquisition buffer [20 mM tris-HCl (pH7.4), 118 mM NaCl, 1.2 mM KH_2_PO_4_, 1.2 mM MgSO_4_, 4.7 mM KCl, and 1.8 mM CaCl_2_] with either 0.0025% LMNG (w/v), 0.00025% CHS tris (w/v), and 0.0025% GDN (w/v) for Cy3B/red or AF546/647-labeled constructs and 0.005% LMNG (w/v), 0.0005% CHS tris (w/v), and 0.005% GDN (w/v) for AF488/647-labeled constructs. Measurements at saturating ligand concentrations were performed at 10 mM Glu, 100 μM LY379268, 100 μM LY341495, and 1 mM DCG-IV either in the absence or presence of 1 μM (AF488/647 constructs) or 10 μM BINA (Cy3B/red and AF546/647 constructs). The addition of BINA resulted in an increased fluorescent background when excited at a wavelength of 488 nm. We found that applying 1 μM kept the fluorescent background low enough to separate it from single-molecule photon bursts of AF488-labeled receptors while still promoting full VFT reorientation (fig. S6F). The heterotrimeric G_i1_ complex was a gift from S. Granier and R. Sounier (IGF Montpellier, France) and was applied at 1 μM final concentration as previously described in (*18*).

### smFRET data analysis

Data analysis was performed using the PAM 1.3 software package (*56*). A single-molecule event was defined as a burst containing at least 40 photons with a maximum allowed interphoton time of 0.16 ms and a Lee-filter of 30. Apparent FRET efficiencies (*E*_PR_), accurate FRET efficiencies (*E*), and stoichiometry (*S*) were calculated as described previously (*18*) following the recommendations made in (*57*).

Values for donor leakage α (fraction of the donor emission into the acceptor detection channel), direct excitation δ (fraction of the direct excitation of the acceptor by the donor-excitation laser), γ (correction factor that considers effective fluorescence quantum yields and detection efficiencies of the acceptor and donor), and β (correction factor that considers the relative laser excitation intensities) were α = 0.22, δ = 0.09 (SNAP-Cy3B/red); α = 0.1, δ = 0.13, γ = 0.7, β = 0.95 (248-AF546/647); α = 0.08, δ = 0.12, γ = 0.9, β = 1.2 (358-AF546/647); and α = 0.02, δ = 0.05, γ = 1.2, β = 0.75 (248 + 358-AF488/647).

To display FRET histograms, doubly labeled molecules with an *S* = 0.3 to 0.7 (*S* = 0.35 to 0.75 for SNAP-Cy3B/red) were selected and normalized for the same number of molecules based on the macrotime of the experiment for individual biological replicates. In this way, average histograms with the mean FRET efficiency ± SEM were obtained. Fitting was performed using Origin 6 (Microcal Software Inc.) on average FRET histograms and for each individual dataset separately to derive data shown in scatterplots. Histograms and plots were displayed using GraphPad Prism 7.05.

### Additional software

The structures shown were generated using PyMOL 2.3.3. Figures were generated using Microsoft PowerPoint 2019 and Inkscape 0.92.

## Supplementary Materials

### This PDF file includes:

Figs. S1 to S12 Table S1 Supplementary Protocol Legend for figures data References

## Notes

View/request a protocol for this paper from *Bio-protocol*.
