# Supplementary material for: Concerted conformational changes control metabotropic glutamate receptor activity
Source: Sci Adv. 2023 Jun 2;9(22):eadf1378. doi: 10.1126/sciadv.adf1378 (PMC10413646; doi:10.1126/sciadv.adf1378)
Supplement: Supplementary file 1 — Figs. S1 to S12 Table S1 Supplementary Protocol Legend for figures data References [file sciadv.adf1378_sm.pdf]

Science Advances
AAAS

Supplementary Materials for

Concerted conformational changes control metabotropic glutamate receptor activity

Nathalie Lecat-Guillet *et al.*

Corresponding author: Emmanuel Margeat, emmanuel.margeat@cbs.cnrs.fr;
Philippe Rondard, philippe.rondard@igf.cnrs.fr

Sci. Adv. **9**, eadf1378 (2023)
DOI: 10.1126/sciadv.adf1378

The PDF file includes:

Figs. S1 to S12
Table S1
Supplementary Protocol
Legend for figures data
References

Other Supplementary Material for this manuscript includes the following:

Figures data

PDB	Subtype	Construct	Ligands	Conformation	PMID
1eWK	mGlu1	ECD	Glutamate (Ago)	Aoc	11069170
1EWT	mGlu1	ECD	-	Roo	11069170
1EWV	mGlu1	ECD	-	Aoc	11069170
1ISS	mGlu1	ECD	S-MCPG (Antagonist)	Roo	11867751
3KS9	mGlu1	ECD	LY341495 (Antago)	Aoo	unpublished
7DGD	mGlu1	Full length	-	Roo	33278019
7DGE	mGlu1	Full length	Quisqualic acid (Ago), Nb43	Aoc	33278019
7E9G	mGlu2	Full length	Gi1, scFV16, Nb13, LY354740(Ago), JNJ-40411813 (PAM)	Acc	34135510
7EPA	mGlu2	Full length	-	Roo	34135509
7EPB	mGlu2	Full length	LY354740 (Ago)	Acc	34135509
7MTQ	mGLu2	Full length	LY341495 (Antagonist)	Roo	34194039
7MTR	mGLu2	Full length	ADX55164 (AgoPAM), Glutamate (Ago)	Acc	34194039
7MTS	mGLu2	Full length	ADX55164 (AgoPAM), Glutamate (Ago), Gi	Acc	34194039
7EPD	mGlu2/7	Full length	-	Roo	34135509
2E4U	mGlu3	ECD	Glutamate (Ago)	Rcc	17360426
2E4V	mGlu3	ECD	DCGIV (Ago)	Rcc	17360426
2E4W	mGlu3	ECD	1S,3S-ACPD (Ago)	Rcc	17360426
2E4X	mGlu3	ECD	1S,3R-ACPD (Ago)	Rcc	17360426
2E4Y	mGlu3	ECD	2R,4R-APDC (Ago)	Rcc	17360426
7WI6	mGlu3	Full length	LY341495 (Antagonist), VU0650786 (NAM)	Roo	35236939
7WI8	mGlu3	Full length	LY341495 (Antagonist)	Roo	35236939
7WIH	mGlu3	Full length	LY2794193 (Ago)	Acc	35236939
7E9H	mGlu4	Full length	Gi3, scFV16, l-serine-O-phosphate (Ago)	Acc	34135510
3LMK	mGLu5	ECD	Glutamate (Ago)	Acc	unpublished
6N51	mGlu5	Full length	Quisqualic acid (Ago) and Nb43	Acc	30675062
6N52	mGlu5	Full length	-	Roo	30675062
7FD8	mGlu5	Full length	Quisqualic acid (Ago)	Acc	34469715
7FD9	mGlu5	Full length	LY341495 (Antagonist)	Roo	34469715
7EPC	mGlu7	Full length		Roo	34135509
6BSZ	mGLu8	ECD	Glutamate (Ago)	Acc	29402739
6BT5	mGLu8	ECD	L-AP4 (Agonist)	Acc	29402739

Supplementary Table 1: Summary of structures used to generate Supplementary Figure 6. Given are the PDB ID, the mGlu subtype, the type of construct being only the extracellular domain (ECD) or full-length receptor, the bound ligand(s), the respective VFT conformation and the PMID of the respective publication.

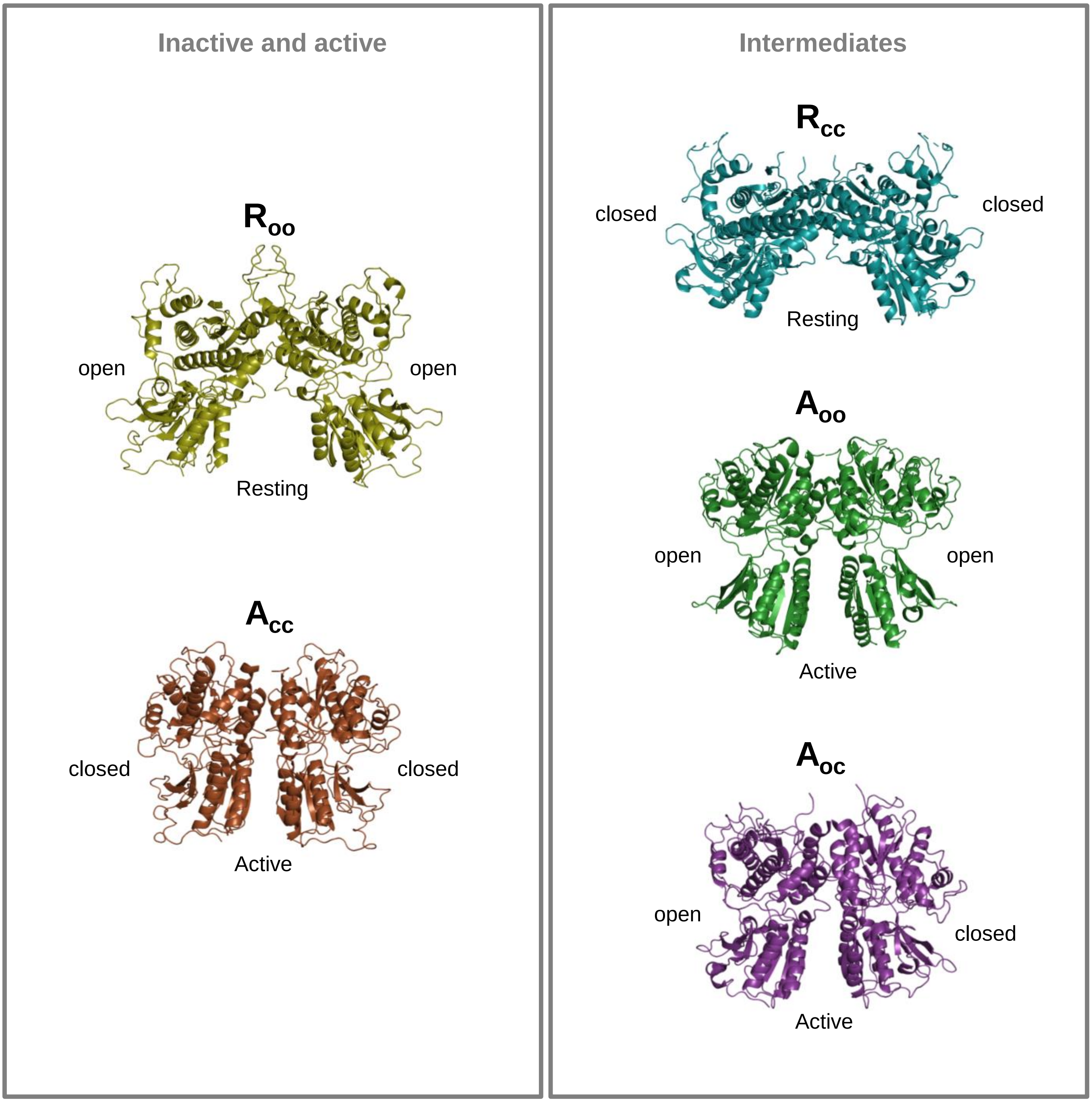

Supplementary Figure 1: Putative conformational states of the mGlu VFT domain proposed from structural studies. Shown are the VFT dimers in inactive Resting open/open (R_{oo}, yellow), Active closed/closed (A_{cc}, brown), Resting closed/closed (R_{cc}, cyan), Active open/open (A_{oo}, green) and Active open/closed (A_{oc}, purple) conformation. R_{oo}: mGlu2, PDB ID 7EPA, amino acids 24-500; A_{cc}: mGlu2, PDB ID 7E9G, amino acids 6-499; R_{cc}: mGlu3, PDB ID 2E4U, amino acids 30-508; A_{oo}: mGlu1, PDB ID 3KS9, amino acids 28-511; A_{oc}: mGlu1, PDB ID 7DGE, amino acids 35-523.

A

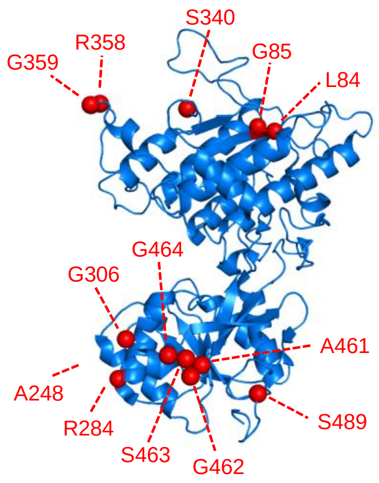

B

Position	VFT Lobe	Yield (% WT)	Functional (IP-1)
84	Upper lobe	21.2 +/-1.1	Yes
85	Upper lobe	23.4 +/-0.7	No
340	Upper lobe	12.3 +/-0.5	Yes
358	Upper lobe	29.8 +/-0.3	Yes
359	Upper lobe	32.6 +/-4.7	Yes
248	Lower lobe	35.9 +/-3.2	Yes
284	Lower lobe	29.5 +/-2.2	Yes
306	Lower lobe	25.5 +/-4.5	Yes
461	Lower lobe	no	NA
462	Lower lobe	no	NA
463	Lower lobe	no	NA
464	Lower lobe	no	NA
489	Lower lobe	no	NA
248/84	Both	5.8 +/-0.7	Yes
248/85	Both	11.8 +/-1.6	No
248/340	Both	15.7 +/-3.5	No
248/358	Both	36.1 +/-1	Yes
248/359	Both	17.1 +/-2	Yes

Supplementary Figure 2: Screening of positions for incorporation of ncAAs. a) Structure of a single VFT (PDB 7EPA) with positions screened for PrF incorporation highlighted in red. b) Summary of screening results showing the positions substituted by PrF, their location within the VFT, their incorporation efficiency relative to expression of the wildtype receptor and their functionality. Expression relative to the wildtype was determined by time-resolved fluorescence measurements of N-terminally labeled SNAP-tags with BG-Lumi4-Tb (no: no surface labeled receptors). Functionality was evaluated by the increase in inositol phosphate accumulation in response to activation with saturating LY379268 as compared to no activation in the absence of ligand using the IP-1 accumulation. Data represent the mean +/-SEM of duplicate analysis. NA: not applicable.

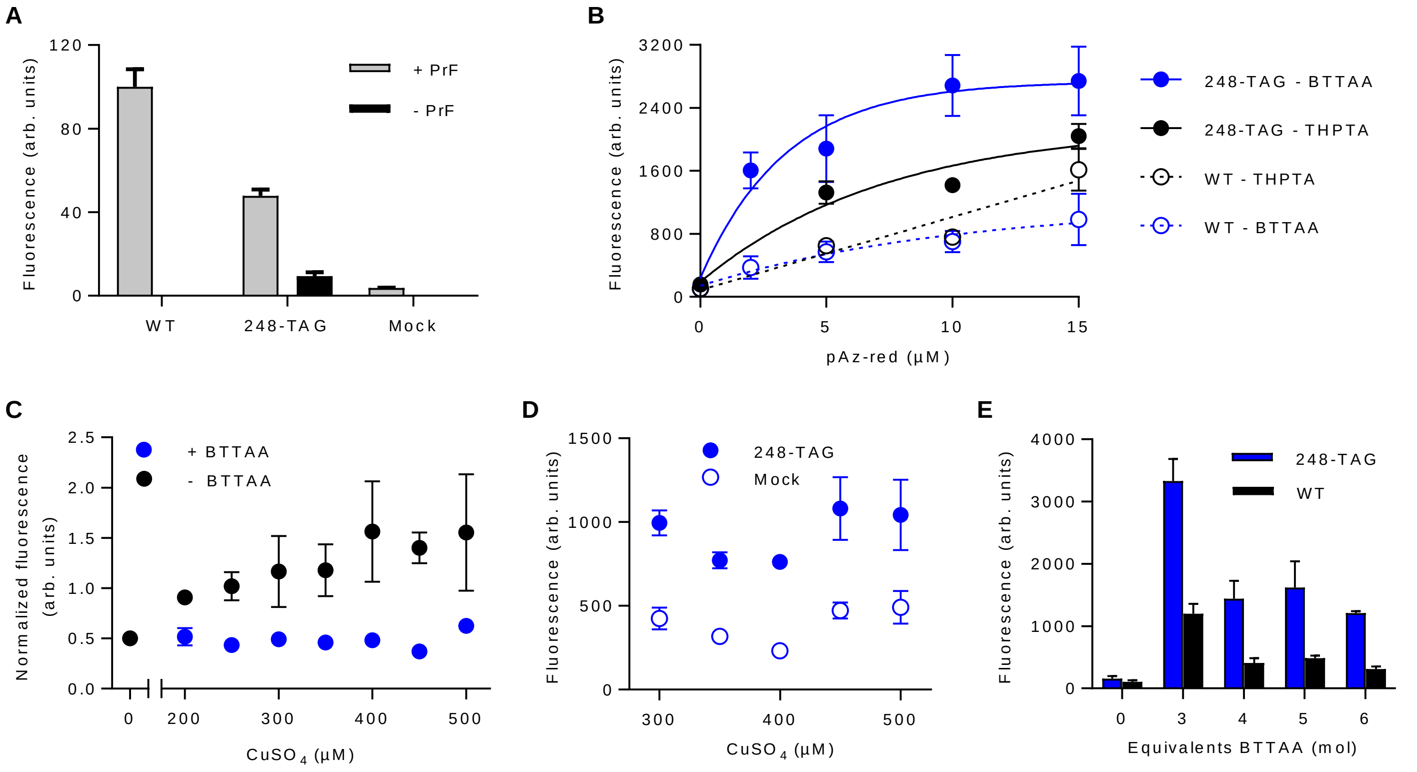

Supplementary Figure 3: Optimization of live cell compatible click chemistry conditions. A) PrF-dependent suppression of premature TAG at position 248 (248-TAG) leads to expression of full-length SNAP-mGlu2-PrF248 as shown by cell surface specific SNAP-labeling with BG-Lumi4-Tb. **B**) Labeling efficiency of CuAAC reaction using BTTAA (blue, 360 µM $CuSO_4$, 6 eq. BTTAA) or THPTA (black, 300 µM $CuSO_4$, 6.6 eq. THPTA). Labeling was performed on cells expressing SNAP-mGlu2-PrF248 or wildtype receptor with 15 µM of pAz-red for 25 min at 37°C. **C**) Cytotoxicity test in response to increasing concentrations of $CuSO_4$ in the absence (black) or presence of 6 eq. BTTAA. Cytotoxicity was determined by the increase in fluorescence as a result of propidium iodide staining, normalized by staining with Hoechst 33342. **D**) Influence of $CuSO_4$ concentration on labeling efficiency using 6 eq. BTTAA and 15 µM pAz-red. **E**) Influence of molar ratio between $CuSO_4$ (360 µM) and BTTAA on labeling efficiency (248-TAG) and specificity (WT) using pAz-red. All data are represented as the mean +/- SEM of triplicates.

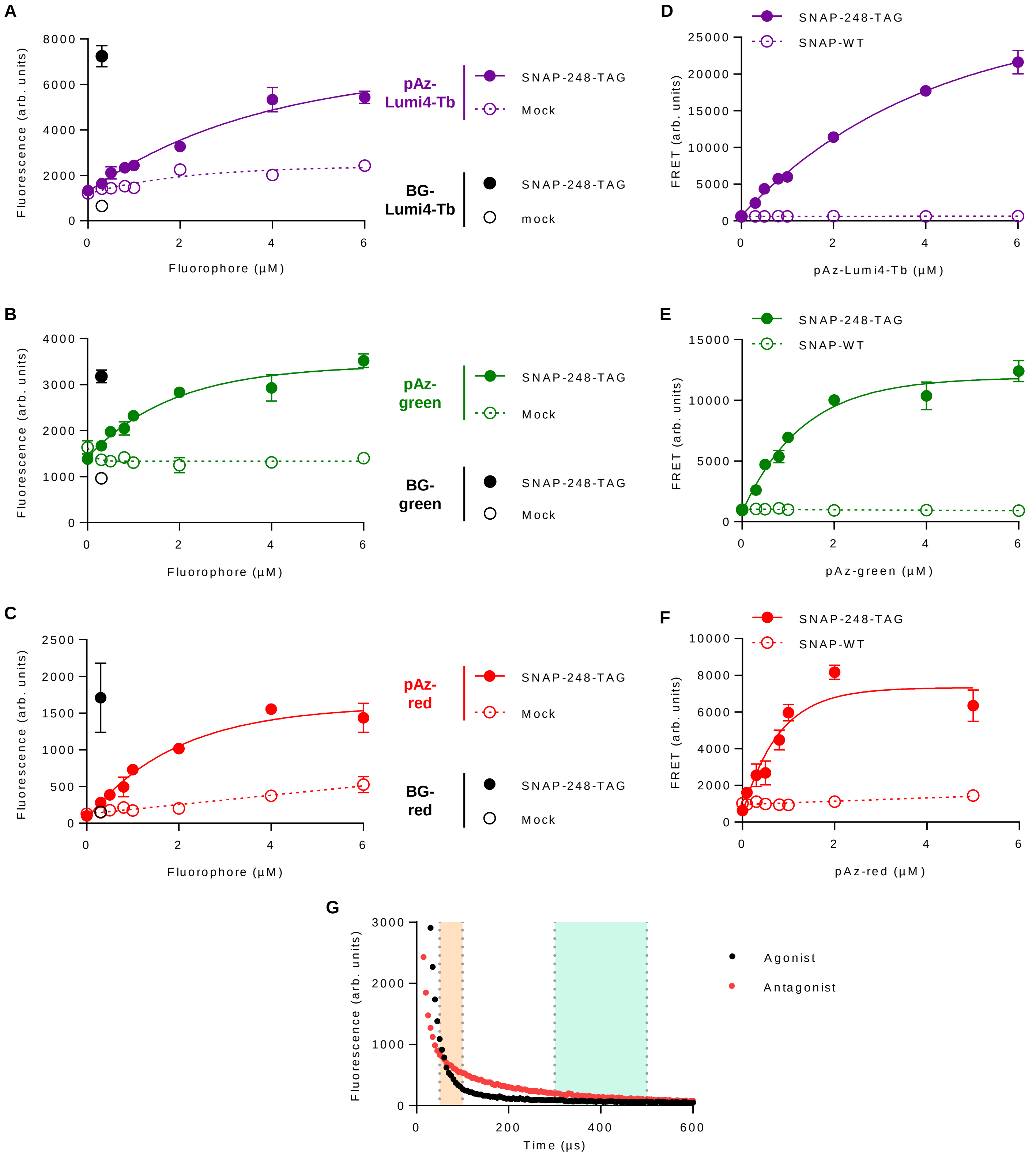

Supplementary Figure 4: Optimization of labeling with pAz dyes for LRET measurements. A-C) Labeling efficiency of SNAP-mGlu2-PrF248 (SNAP-248-TAG, red solid line) and specificity compared to cells transfected with empty vector (Mock, red dashed line) using CuAAC at increasing concentrations of indicated pAzF dyes. Maximal and non-specific labeling of SNAP-tag with corresponding BG-dye derivatives is also shown. **D-F**) Increase in FRET signal in response to labeling with increasing concentrations of pAz dyes. The FRET signal is given as sensitized emission of acceptor after Lumi4-Tb donor excitation by LRET. Labeling was performed with BG-green (**D**) or BG-Lumi4-Tb (**E-F**) followed by CuAAC with pAz-Lumi4-Tb (**D**), pAz-green (**E**) or pAz-red (**F**). All data are represented as the mean +/- SEM of triplicates. **G**) Fluorescence decay of SNAP-248PrF labeled with pAz-green acceptor after excitation of SNAP labeled Lumi4-Tb donor in the presence of 10 µM LY379268 (black) and 100 µM LY341495 (red). The windows used for calculation of the sensitized acceptor emission ratio are indicated (50-100 µs and 300-500 µs).

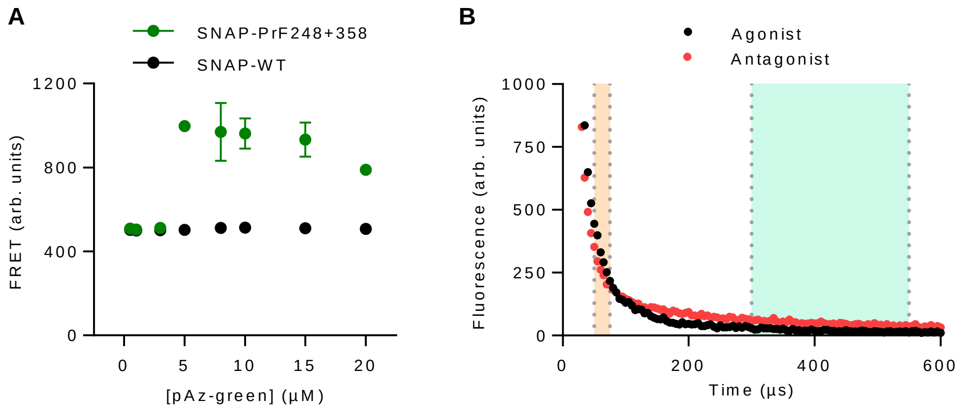

Supplementary Figure 5: Double labeling optimization. A) Optimization of labeling ratio of pAz-Lumi4-Tb and pAz-green. The pAz-Lumi4-Tb concentration was fixed at 3 µM and labeling reactions performed at increasing concentrations of pAz-green. **B**) Fluorescence decay of "green" acceptor after donor excitation of SNAP-PrF248+358 labeled with pAzF-green acceptor and pAz-Lumi4-Tb donor in the presence of 10 µM LY379268 (black) and 100 µM LY341495 (red). The windows used for calculation of the sensitized acceptor emission ratio are indicated (50-75 µs and 300-550 µs).

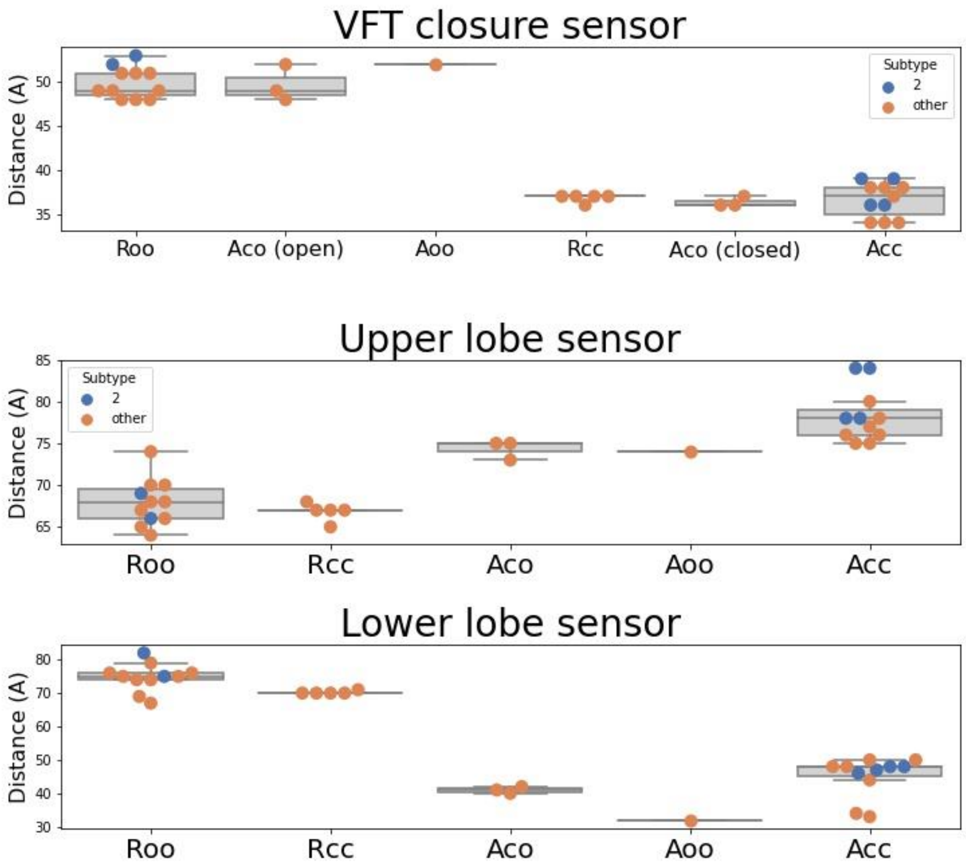

Supplementary Figure 6: Measured distances between Cα of the residues modified in this study to generate the conformational sensors (A248 and F358 in mGlu2 or their equivalents in other mGlu subtypes). Distances corresponding to the full length mGlu2 structures are represented in blue. All the PDB files used are given in supplementary table 1, and correspond to 7 different subtypes (including 1 heterodimer), and full length or isolated ECD structures). It appears that: 1/ The VFT closure sensor will report on the open/closed transition through a low FRET to high FRET transition. 2/ The upper lobe sensor will report on the dimer reorientation from the resting to the active state through a high FRET to low FRET transition. If significantly populated, an A_{co} or A_{oo} conformations might appear at an intermediate FRET value. 3/ The lower lobe sensor will report on the dimer reorientation from the resting to the active state through a low FRET to high FRET transition. If significantly populated, an R_{cc} intermediate conformation might appear at a slightly higher FRET value than the R_{oo} conformation, and an A_{oo} intermediate conformation might appear at a slightly higher FRET value than the A_{cc} conformation.

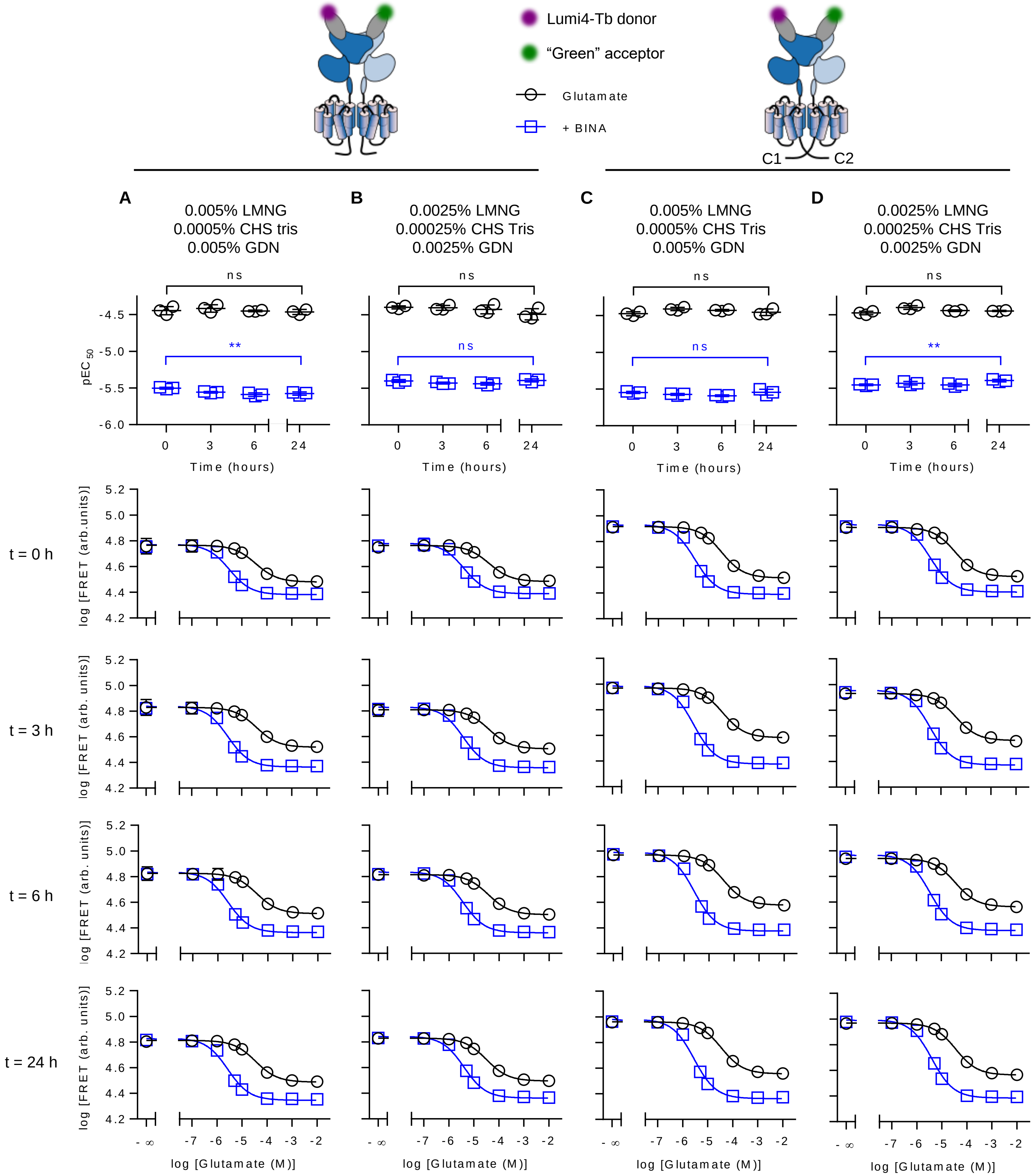

Supplementary Figure 7: Evaluation of functional integrity of receptors over time after solubilization using LRET. Wildtype receptor homodimers (**A-B**) or heterodimers containing the engineered C-terminal C1 and C2 $GABA_B$ quality control system (**C-D**) were labeled with BG-Lumi4-Tb donor and BG-green acceptor via N-terminal SNAP-tags and solubilized from membrane fractions using 1% LMNG + 0.1% CHSTris. Subsequently, supernatants were diluted in the presence of GDN to reach final concentrations of either 0.005% LMNG + 0.0005% CHS Tris + 0.005% GDN (**A** and **C**) or 0.0025% LMNG + 0.00025% CHS Tris + 0.0025% GDN (b and d) in acquisition buffer and titrated with glutamate alone (black) or glutamate + 10 µM BINA (blue). Functional integrity over time at room temperature was evaluated based on the changes in glutamate and glutamate + BINA pEC_{50} values (top row) obtained from dose-response curves performed on three independent biological replicates analyzed in one experiment and are presented together with the mean +/- SEM. Statistical differences were determined using two-sided, unpaired t-tests and are given $^{**}p \leq 0.01$, ns > 0.05. The average dose-response curves were obtained by fitting the mean +/- SEM at t = 0, 3, 6 and 24h of sample storage at room temperature (from top to bottom) are shown below the corresponding pEC_{50} plots with.

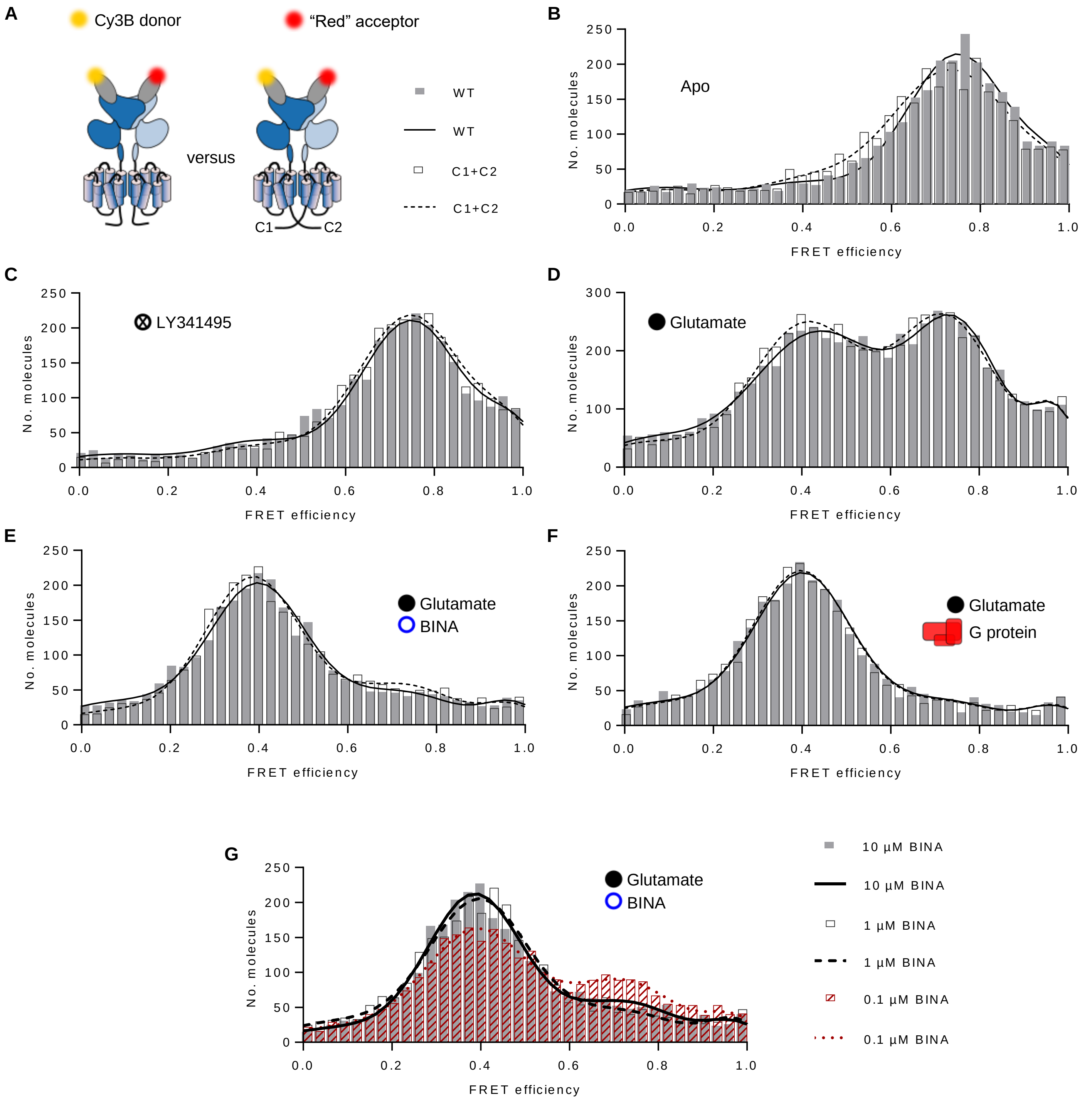

Supplementary Figure 8: Comparison of ligand-induced VFT reorientation between wildtype and C-terminally modified mGlu2. Receptor constructs were labeled with BG-Cy3b donor and BG-D2 acceptor on N-terminal SNAP-tags, solubilized and VFT reorientation measured by smFRET at different ligand conditions. **A**) Shown are representative FRET histograms of wildtype receptor (WT, gray bars, global fit given as black solid line) superimposed on histograms obtained for C-terminally modified receptor (C1+C2, white bars, global fit given as black dashed line) in the absence of ligand (**B**, Apo), the presence of saturating LY341495 (**C**), glutamate (**D**), glutamate + BINA (**E**) and glutamate + heterotrimeric G_i protein (**F**). **G**) Superimposition of representative FRET histograms obtained for C-terminally modified SNAP-mGlu2, labeled as above, in the presence saturating glutamate together with 0.1 µM BINA (red bars with global fit given as red dashed line), 1 µM BINA (white bars with global fit given as black dashed line) and 10 µM BINA (gray bars with global fit given as solid black line). All histograms were obtained by plotting a fixed number of doubly labeled molecules (S = 0.35-0.75, a-b and d-f = 3000+/-2 molecules, c = 6000+/-2 molecules) versus the FRET efficiency given as E_{PR} (corrected for direct excitation and crosstalk as specified in Methods).

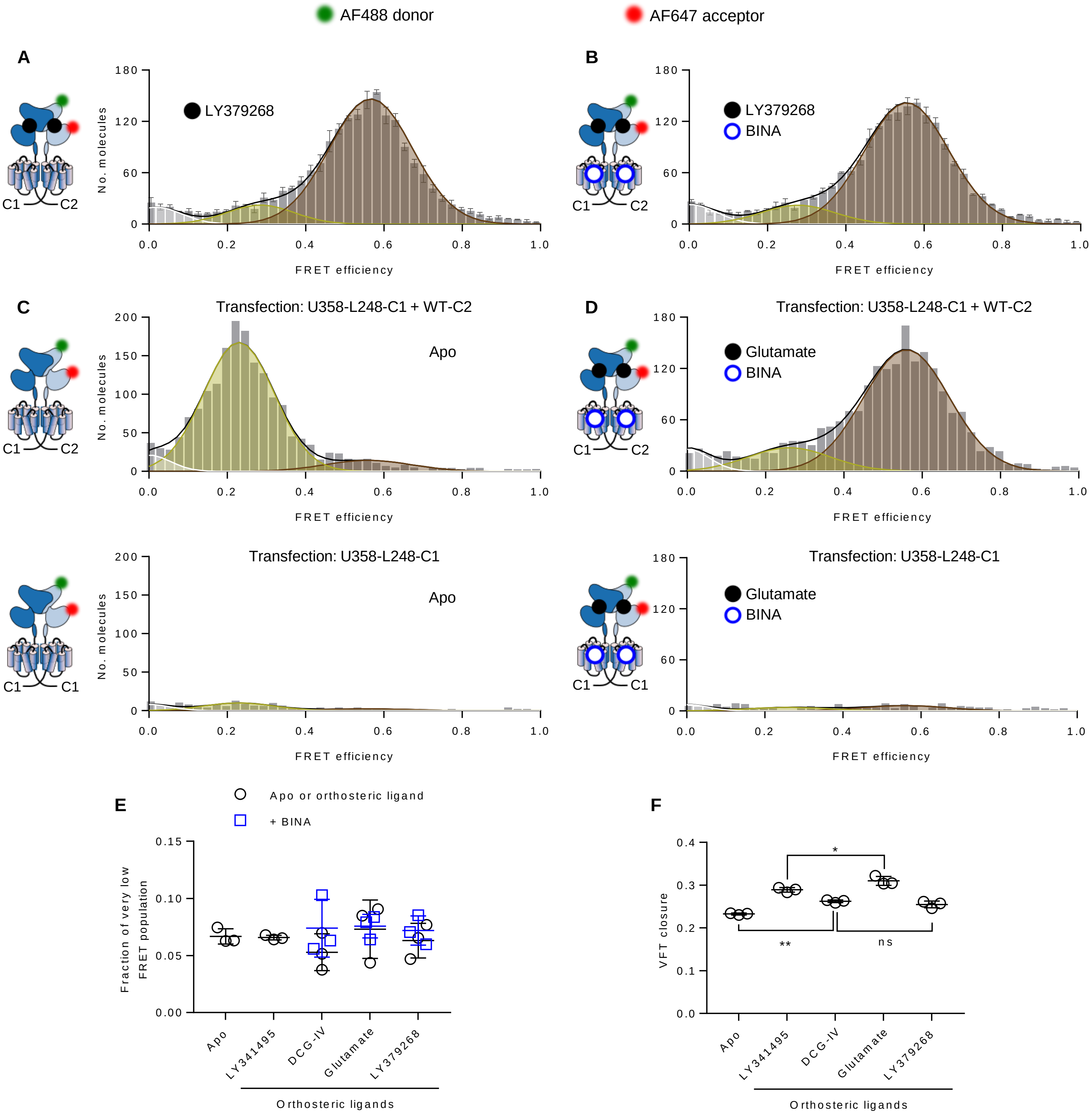

Supplementary Figure 9: Supplementary data for VFT closure sensor. FRET histograms of VFT closure sensor (U_{358}-L_{248}) in the presence of a saturating 100 µM LY379268 (**A**) and 100 µM LY379268 + 1 µM BINA (**B**). FRET histograms show the accurate FRET efficiency as the mean +/- SEM of three independent biological replicates each normalized to 2000 events in the DA population (S = 0.3-0.7). Histograms display the fitting with 3 gaussians (white = very low FRET, yellow = low FRET, brown = high FRET) together with the global fit (black). **C-D**) Representative FRET histograms of samples obtained by expression of 248TAG+358TAG-C1 in the presence (top) or absence (bottom) of WT-C2. Top and bottom histograms in the absence (**C**) or presence of glutamate + 1 µM BINA (**D**) were each plotted for the same measurement macrotime and corrected for the applied sample volume using samples that were prepared in the same manner in parallel (**C**: 8955 s at 16 µl = 4776 s at 30 µl, **D**: 11620 s at 13 µl = 5035 s at 30 µl) to evaluate the degree of signal obtained from leakage of the 248+358-C1 sensor to the cell surface in the absence of WT-C2. **E**) Representation of the fraction of population of the very low state in response to different ligand conditions given as the number of molecules found in the respective population over the sum of molecules in all three populations. Black circles show the Apo condition and orthosteric ligands, while blue squares are given for agonists in the presence of 1 µM BINA. **F**) Determination of the mean FRET efficiency of the low FRET (yellow) population (VFT closure) for the different ligand conditions shown in Fig. **3B-E** and **S9A**. Data in **E-F** are given with the mean +/- SEM of three independent biological replicates. **F**) Statistical differences were determined using a one-way ANOVA with Tukey's multiple comparisons test and are given as ****p ≤ 0.0001, ***p ≤ 0.001, **p ≤ 0.01, *p ≤ 0.05, ns > 0.05.

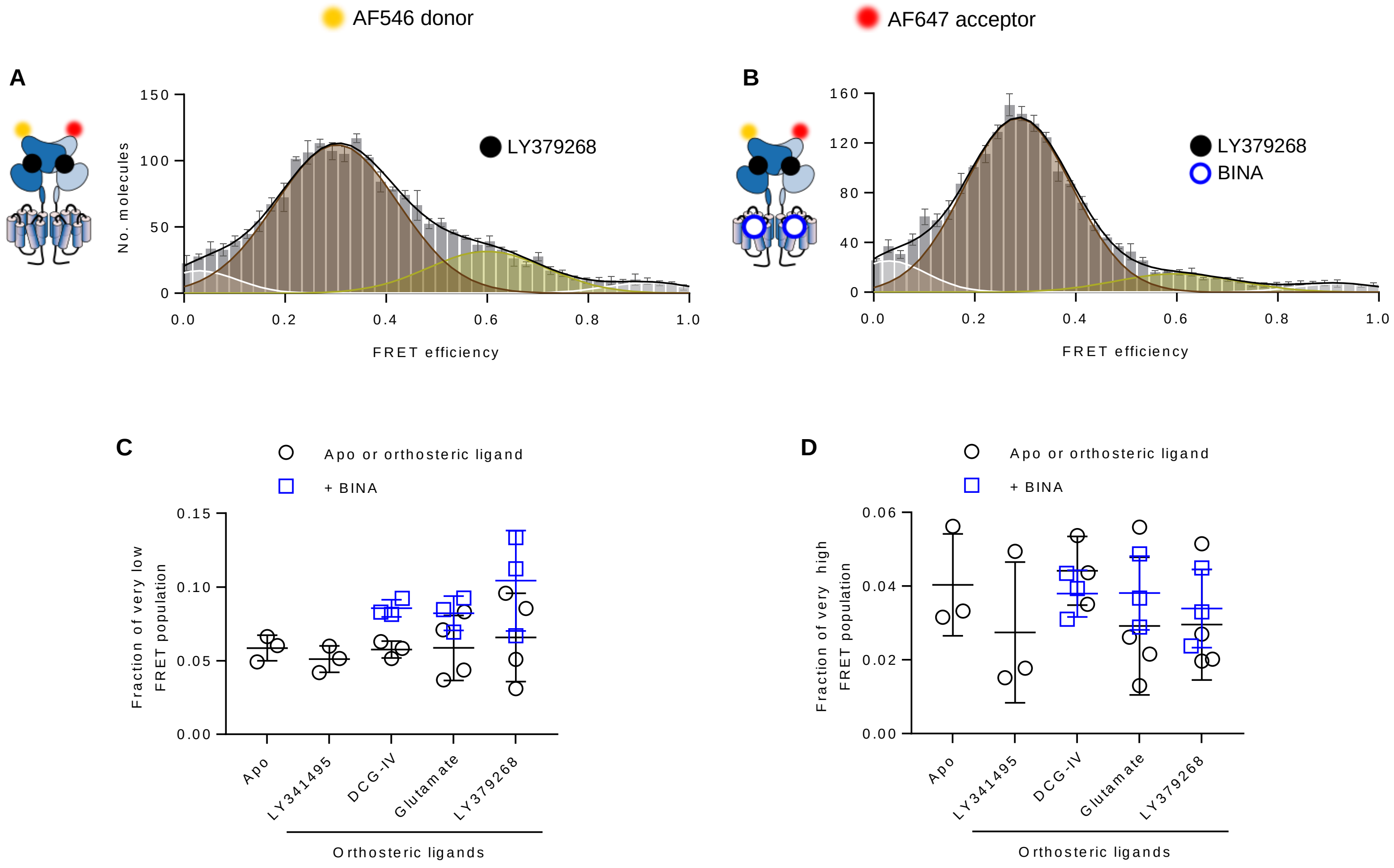

Supplementary Figure 10: Supplementary data for upper lobe FRET sensor. A-B) FRET histograms of upper lobe sensor (U_{358}-U_{358}) in the presence of a saturating 100 µM of the synthetic full agonist LY379268 alone (**A**) and in the presence of 10 µM BINA (**B**).Histograms show the accurate FRET efficiency as the mean +/- SEM of three independent biological replicates each normalized to 2000 events in the DA population (S = 0.3-0.7). Histograms display the fitting with 4 gaussians (yellow = LF, red = HF, white = VLF and VHF) together with the global fit (black). **C-D**) Representation of the fraction of population of the very low (**C**) and very high (**D**) FRET states in response to different ligand conditions given as the number of molecules found in the respective population over the sum of molecules in all four populations. Black circles show the Apo condition and orthosteric ligands while blue squares are given for agonists in the presence of 10 µM BINA.

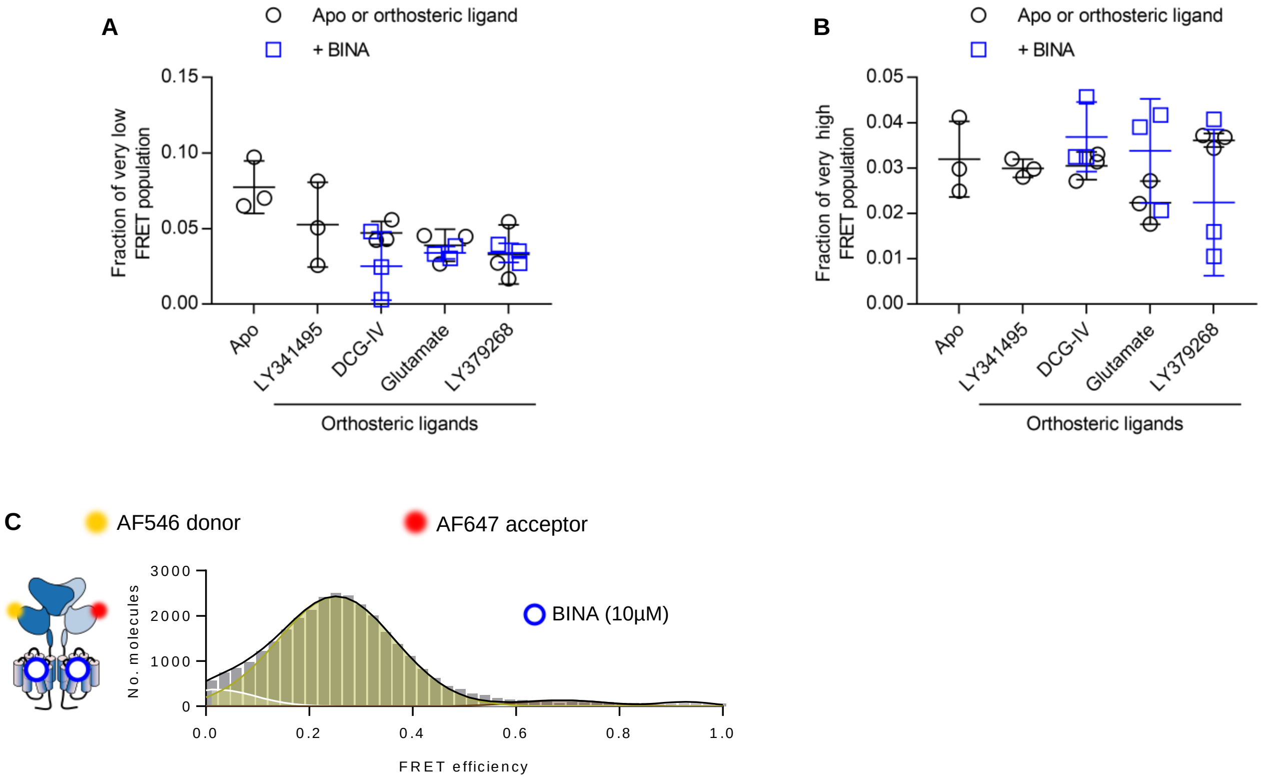

Supplementary Figure 11: Supplementary data for lower lobe FRET sensor. A-B) Representation of the fraction of population of the very low (l) and very high (m) FRET states in response to different ligands, given as the number of molecules found in the respective population over the sum of molecules in all four populations shown in **Fig. 4B-I**. Black circles show the Apo condition and orthosteric ligands, while blue squares are given for agonists in the presence of 10 µM BINA. Data represent the mean +/- SEM of three independent biological replicates. **C**) Representative FRET histograms in the presence of 10 µM BINA.

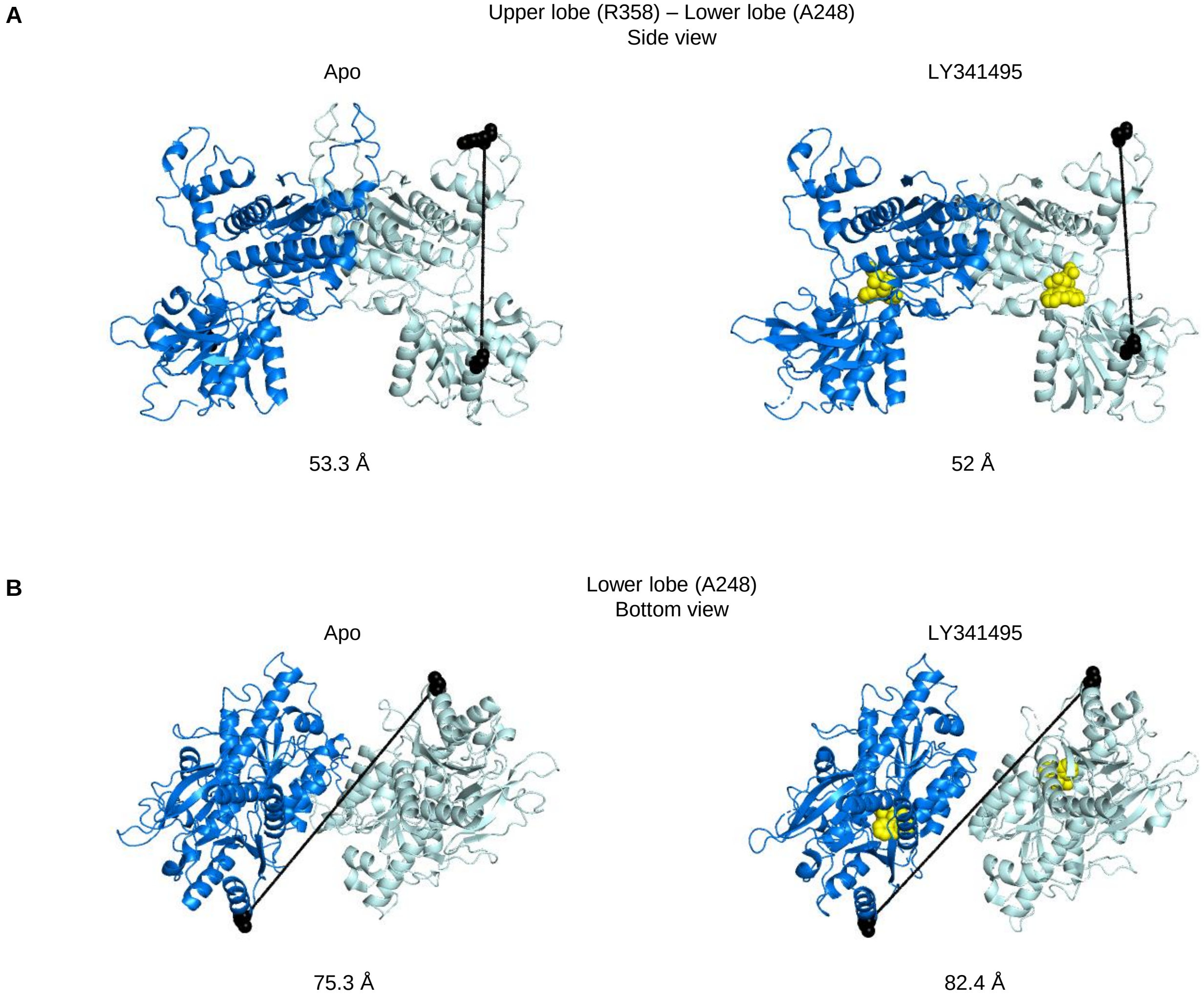

Supplementary Figure 12: Structural differences between apo and antagonist-bound VFTs. Shown are the VFT domains from PDB ID 7EPA (apo) and 7MTQ (LY341495) structures obtained by cryo-EM including distance measurements between the C_α-atoms corresponding to the residues used for the VFT closure (**A**) and lower lobe sensors (**B**).

SUPPLEMENTARY PROTOCOL

Synthesis of picolyl azide fluorophore conjugates

1) General and analytical conditions

pAz-fluorophore compounds have been synthesized according to the following scheme. Lumi4-Tb-NH_2 was obtained according to the synthesis described earlier (58). Alternatively, it can be obtained from PerkinElmer (Codolet, France). Fluoresceine-NH_2 was commercially available from Thermofisher. DY647-NH_2 was commercially available from Dyomics. pAz acid has been synthesized using the procedure described elsewhere (59).

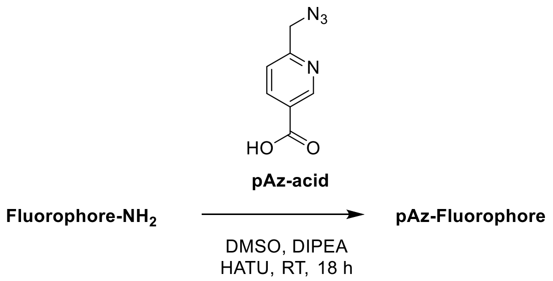

LC-LRMS

LCMS have been run using UHPLC Acquity H Class equipped with Water SQD2 apparatus simple quadripole by electrospray ionization and PDA diode array detector.
LCMS conditions: Column Water Acquity C_{18} 1.7 µm – 2.1 x 50 mm
Flow rate: 0.6 mL.min^{-1}
Gradient : t = 0 : 2 % B – t = 7.5 min : 40 % B
Combinaison solvent for **pAz-red** et **pAz-green**: A: H_2O 0.1 % HCO_2H – B: MeCN 0.1 % HCO_2H
Combinaison solvent for **pAz-Lumi4-Tb**: A: H_2O 5 mM ammonium acetate buffer pH 5.5 – B MeCN

HPLC purification

Purification have been carried out using UHPLC Thermoscientific Dionex Ultimate 3000 using a diode array detector.
Column Water X-Bridge C_{18}– 10 x 100 mm
Flow rate: 4.7 mL.min^{-1}
Gradient solvent for **pAz-red** et **pAz-green**: t = 0 : 5 % B – t = 19 min : 40 % B
Combinaison solvent for **pAz-red** et **pAz-green**: A : H_2O 0.1 % HCO_2H – B : MeCN 0.1 % HCO_2H

Gradient for **pAz-Lumi4-Tb** : t = 0 : 2 % B – t = 19 min : 40 % B
Combinaison solvent for **pAz-Lumi4-Tb** : A : H_2O 25 mM TEAAc buffer pH 7 – B MeCN

HRMS

The analyses were carried out with SYNAPT G2 HDMS mass spectrometer (Waters) equipped with a pneumatically assisted atmospheric pressure (API) ionization source. The sample was ionized in positive electrospray mode under the following conditions: voltage electrospray 2.8kV, orifice voltage: 20V. Nebulization gas flow rate (nitrogen): 100 L.h$^{-1.}$
The high-resolution (MS) mass spectrum was obtained with time-of-Flight (TOF) analyzer. The exact mass measurement was performed in triplicate with double internal calibration.

2) Experimental procedures

pAz-Lumi4-Tb

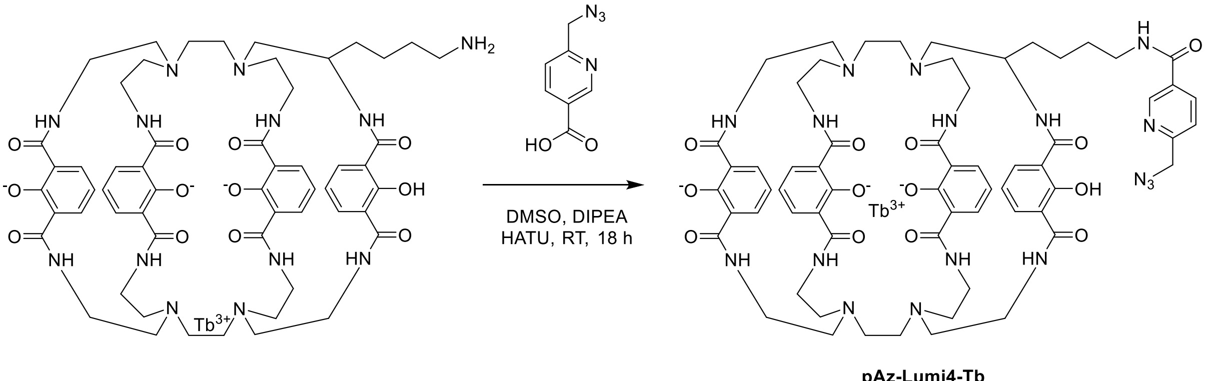

Lumi4-Tb-NH$_2$ **pAz-Lumi4-Tb**

To a solution of the 6-azidomethylnicotinic acid (3.07 mg, 17 µmol) in DMSO (1.44 mL) was added DIEA (18.03 µL, 104 µmol) followed by HATU (6.6 mg, 17 µmol). The mixture was stirred at RT for 15 min. To this mixture was added a solution of Lumi4-Tb-NH$_2$ (20 mg, 16 µmol). The reaction mixture was stirred at RT for 18 h. After this period, the solution was directly purified by semi-preparative HPLC to lead to the desired molecule in 10.5% yield. ESI-LRMS (+) *m/z* 1436 $[M+H]^+$; ESI-HRMS (+) calcd for $[C_{63}H_{74}N_{17}O_{13}Tb +2H]^{2+}$ 718.7531, found 718.7527.

pAz-green

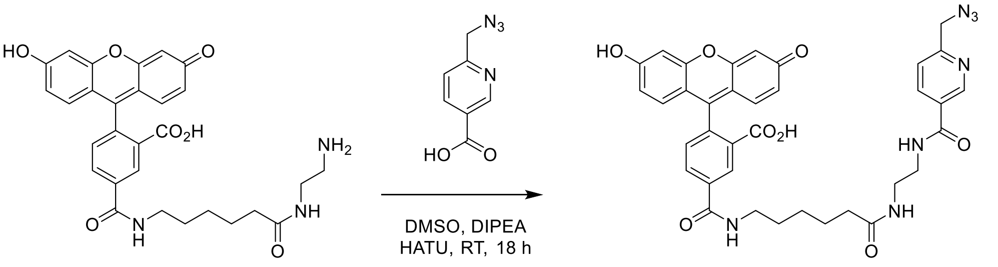

fluoresceine-NH$_2$ **pAz-green**

To a solution of the 6-azidomethylnicotinic acid (1.62 mg, 9 µmol) in DMSO (0.76 mL) was added DIEA (9.5 µL, 55 µmol) followed by HATU (3.5 mg, 9 µmol). The mixture was stirred at RT for 15 min. To this mixture was added a solution of fluorescein-NH$_2$ (5 mg, 8 µmol). The reaction mixture was stirred at RT for 18 h. After this period, the solution was directly purified by HPLC to lead to the desired molecule in 12.9% yield. ESI-LRMS (+) *m/z* 692 $[M+H]^+$; ESI-HRMS (+) calcd for $[C_{36}H_{33}N_7O_8 +H]^+$ 692.2469, found 692.2466.

pAz-red

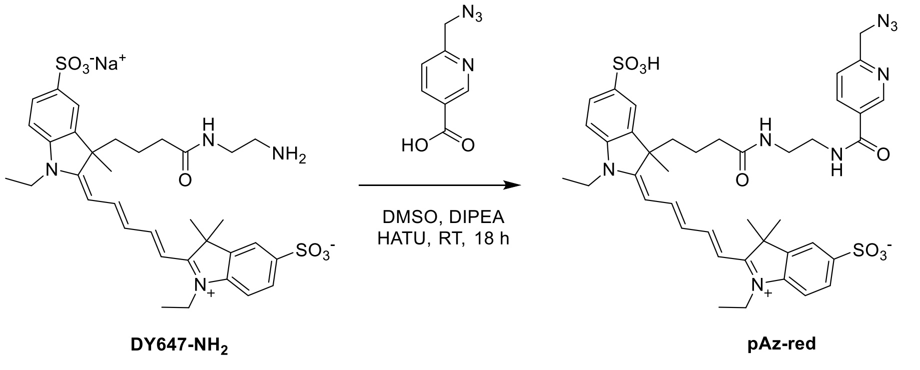

DY647-NH$_2$ **pAz-red**

To a solution of the 6-azidomethylnicotinic acid (1.43 mg, 8 µmol) in DMSO (0.67 mL) was added DIEA (8.4 µL, 48 µmol) followed by HATU (3.1 mg, 8 µmol). The mixture was stirred at RT for 15

min. To this mixture was added a solution of DY647-NH_2 (5 mg, 7 µmol). The reaction mixture was stirred at RT for 18 h. After this period, the solution was directly purified by HPLC to lead to the desired molecule in 54.6% yield. ESI-LRMS (+) *m/z* 845 $[M+H]^+$; ESI-HRMS (+) calcd for $[C_{41}H_{48}N_8O_8S_2+H]^+$ 845.3115, found 845.3110.

3) Chromatograms and mass spectra

pAz-Lumi4-Tb HPLC trace

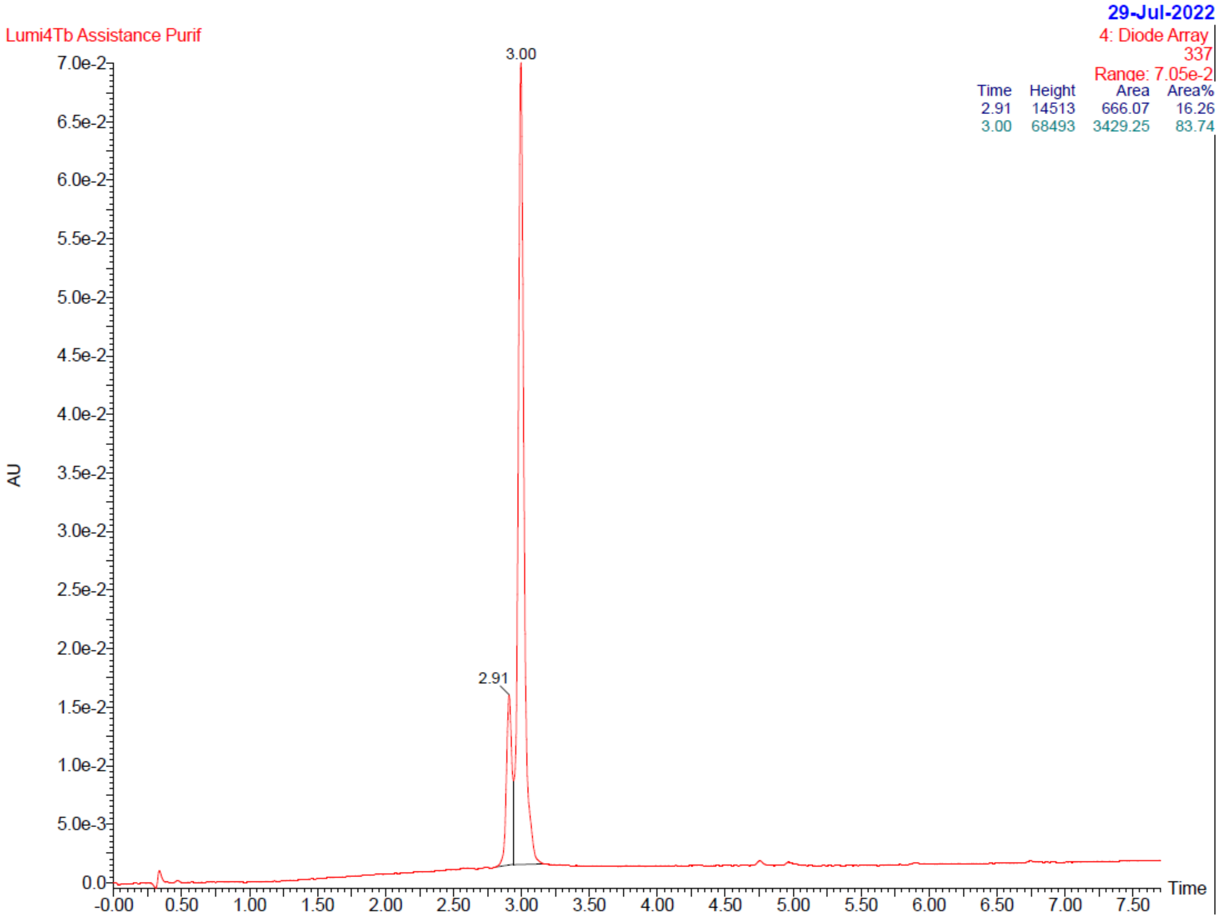

pAz-Lumi4-Tb LRMS in positive mode

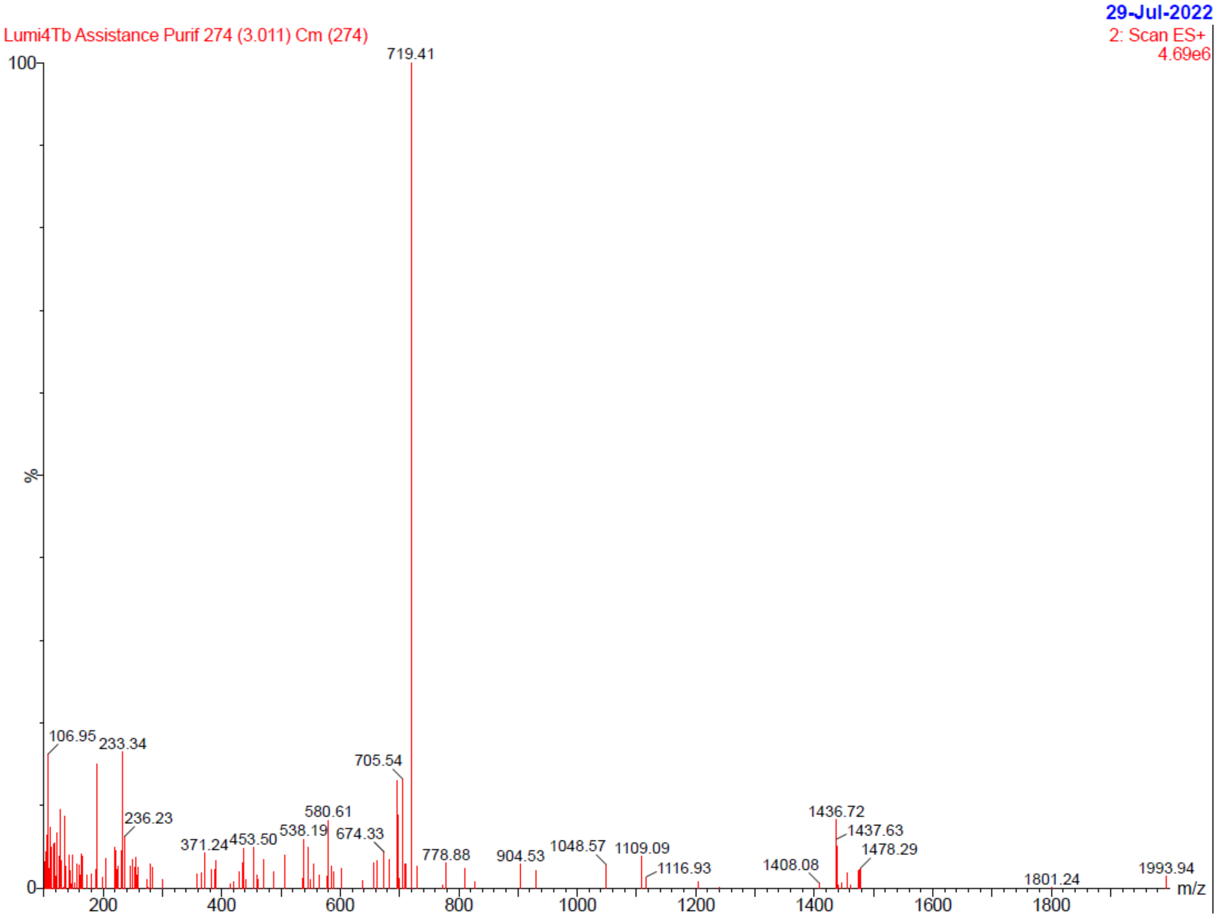

pAz-Lumi4-Tb HRMS

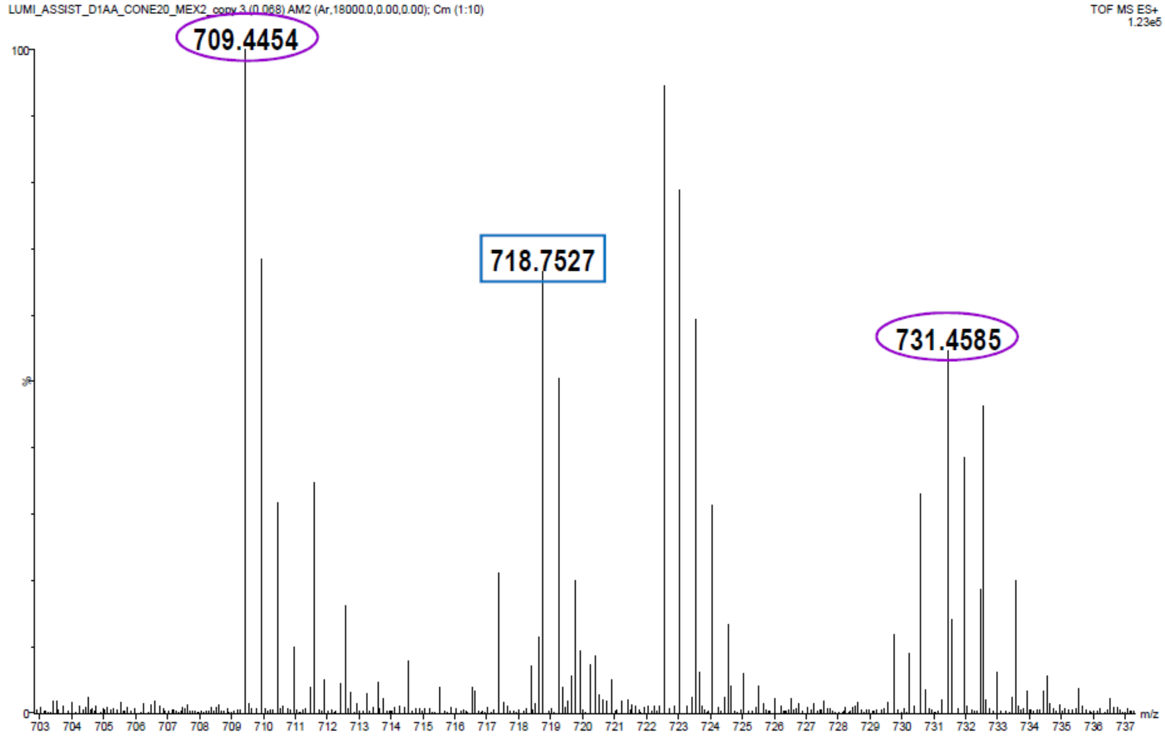

pAz-green HPLC trace

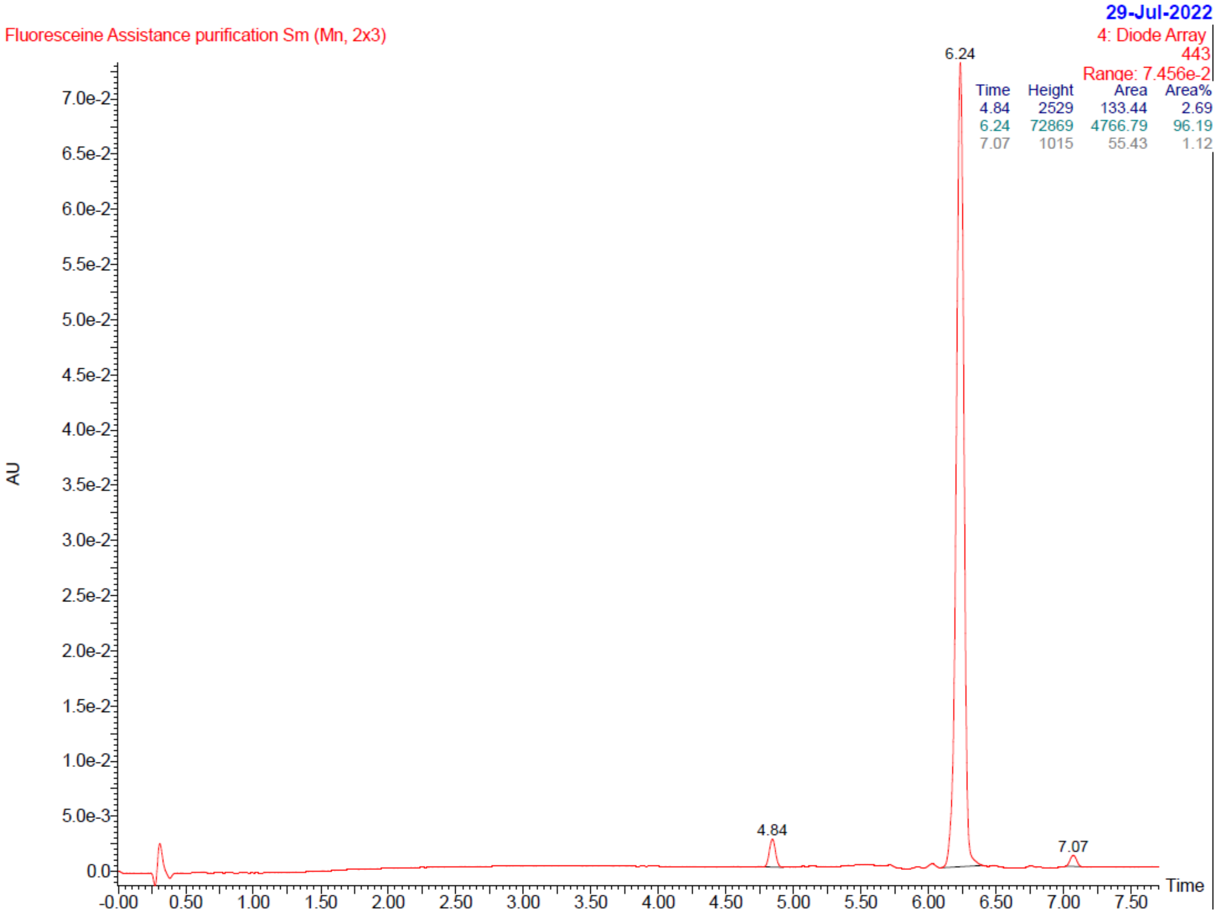

pAz-green LRMS in positive and negative mode

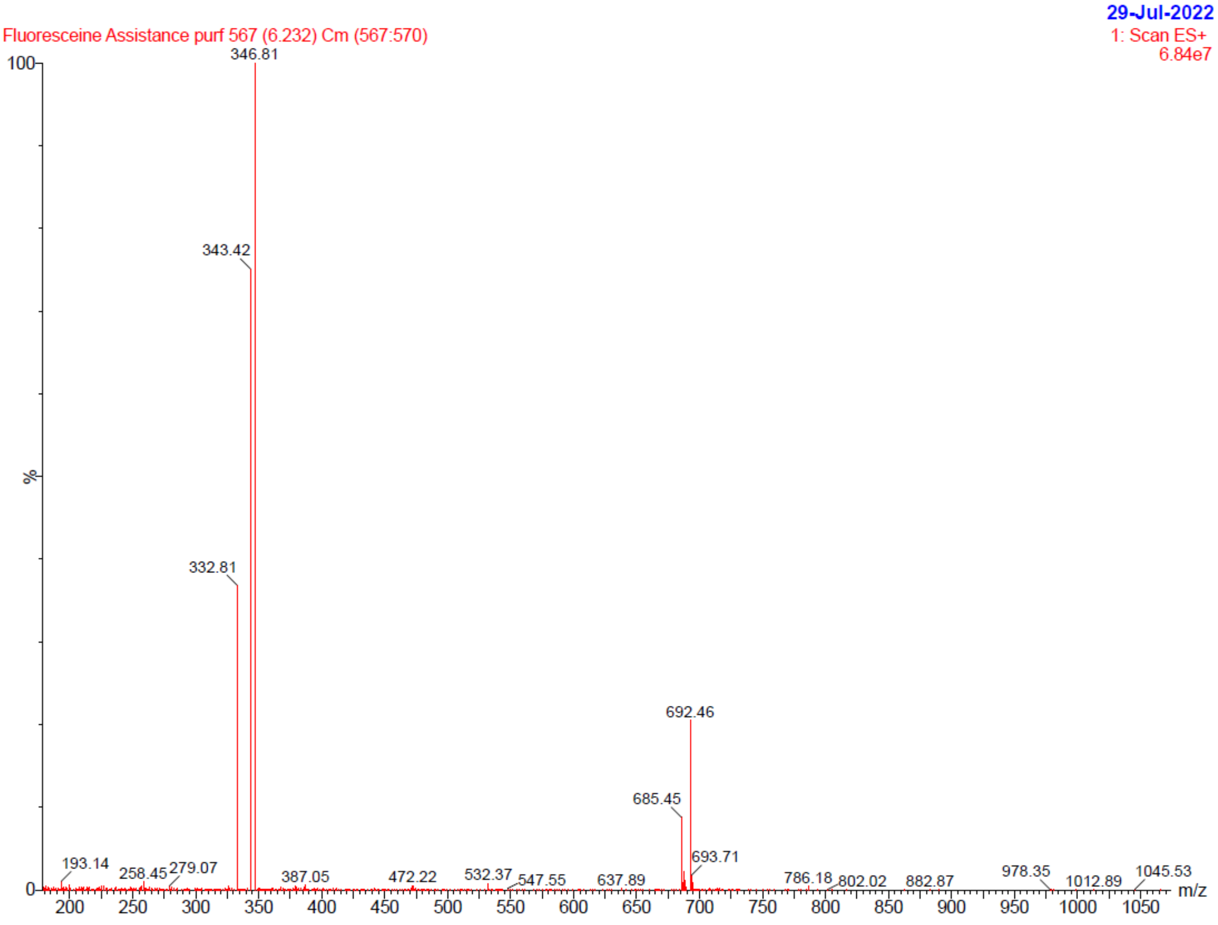

pAz-green HRMS

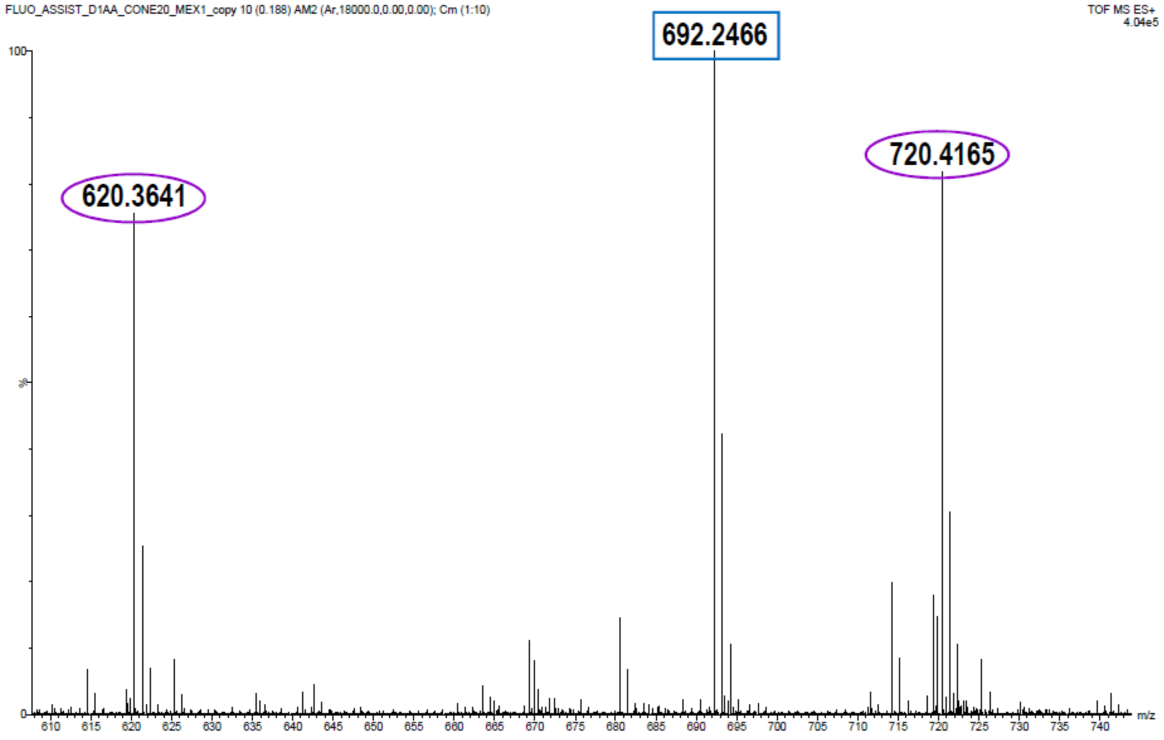

pAz-red HPLC trace

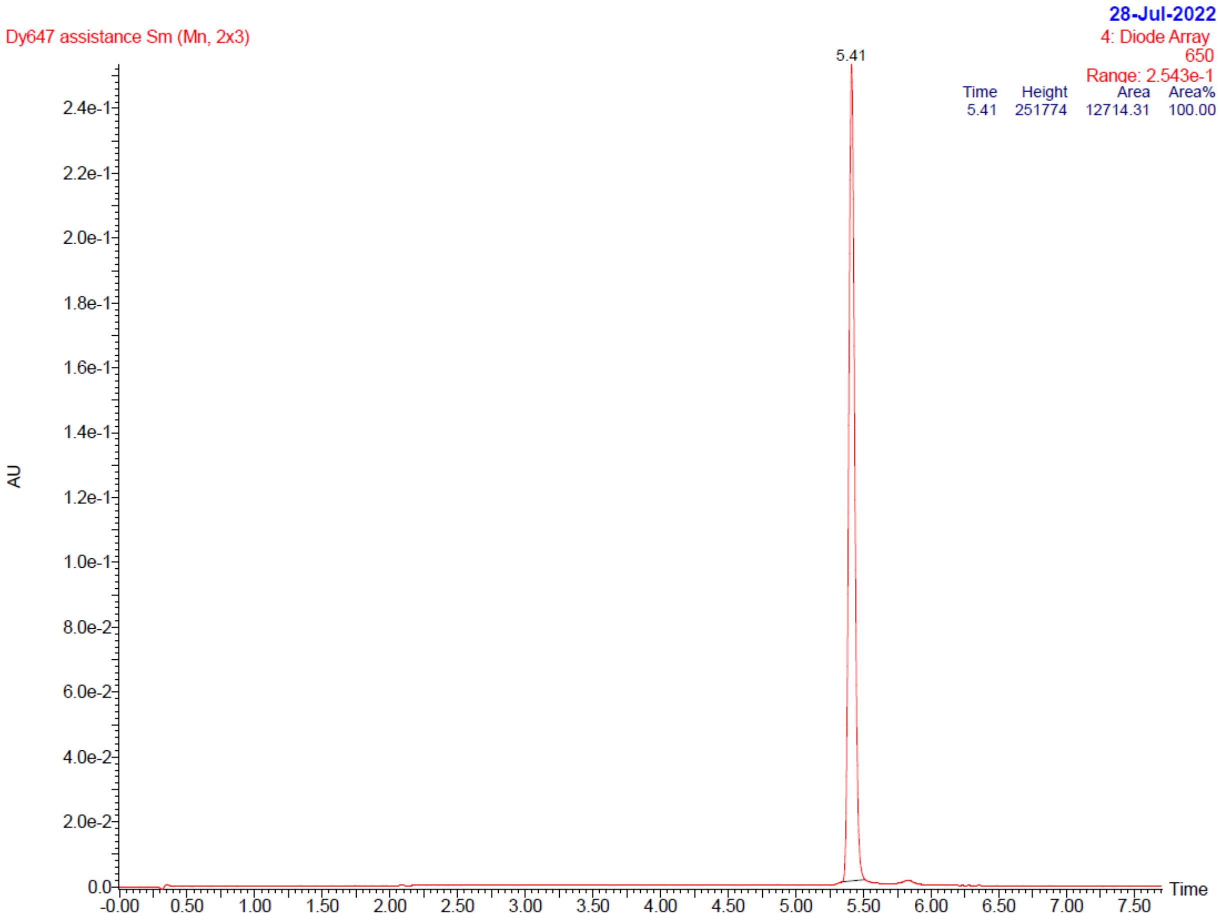

pAz-red LRMS in positive and negative mode

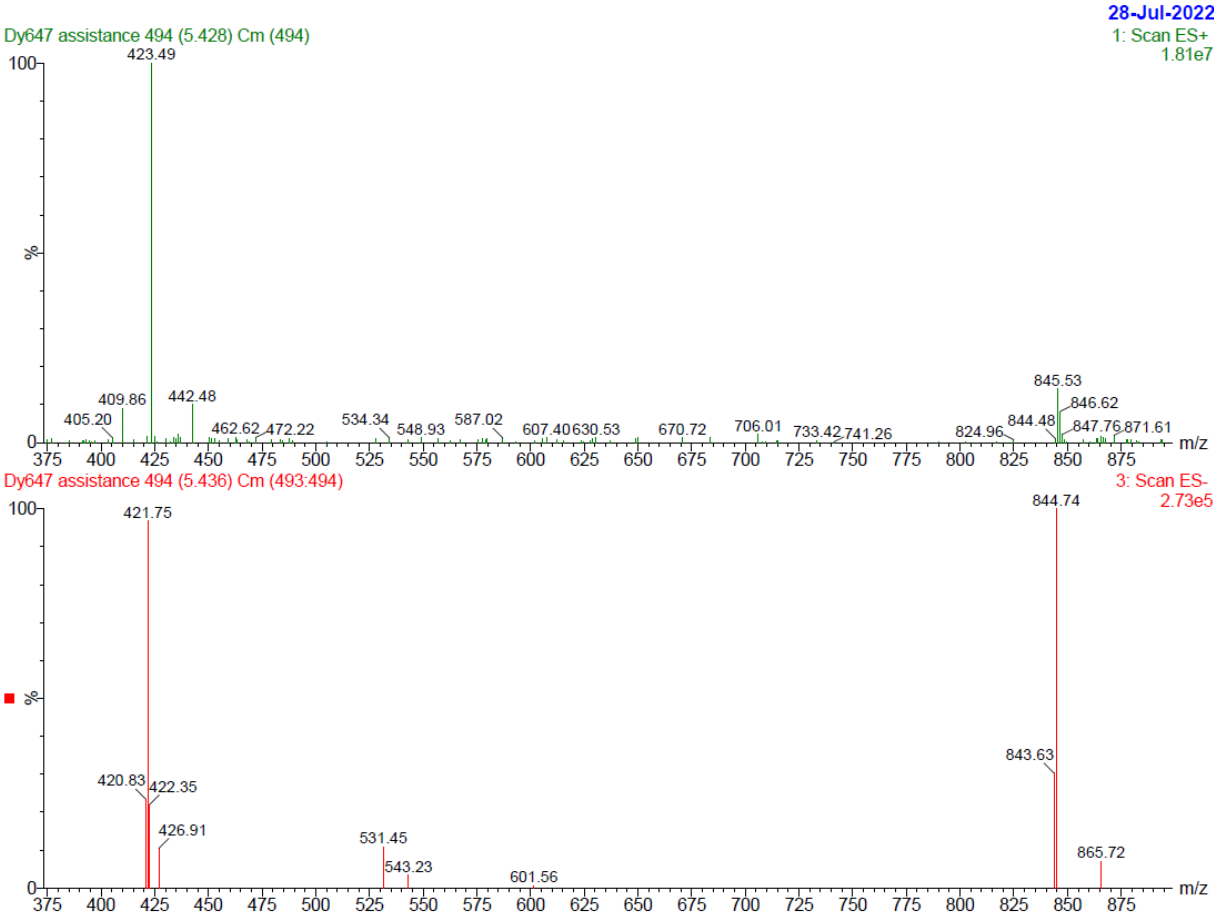

pAz-red HRMS

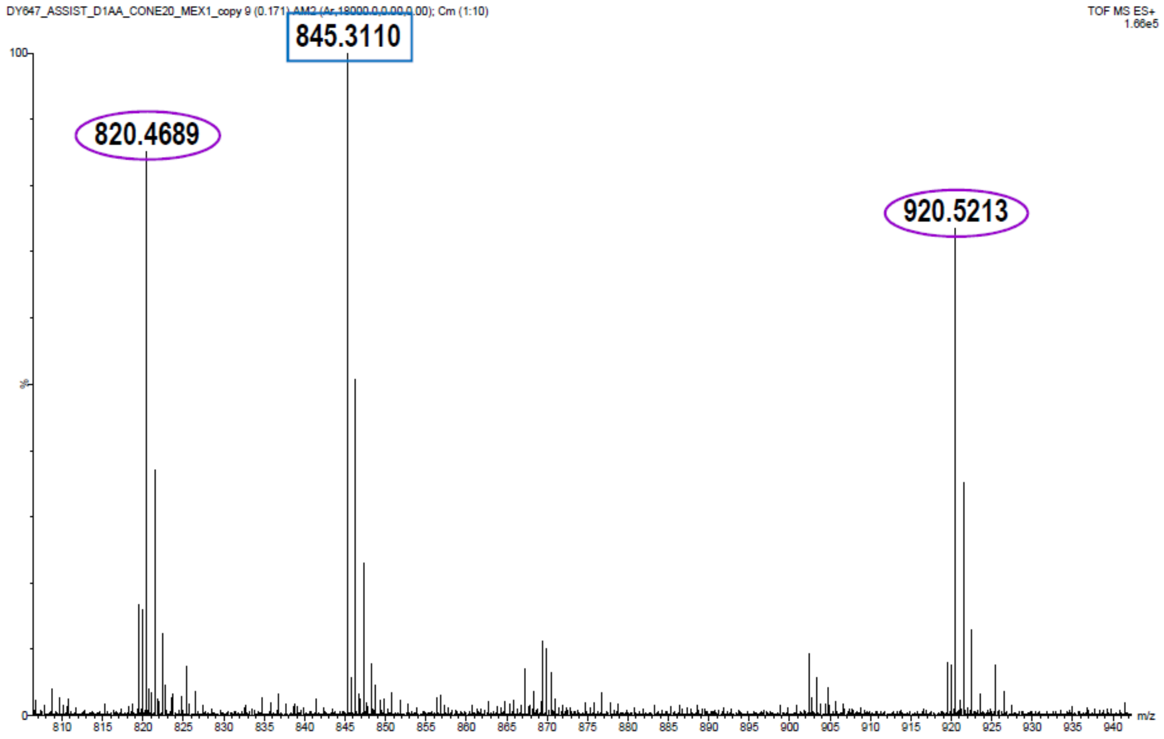

FIGURES DATA

An Excel file containing the data used to generate all Figures is provided as an additional supplementary document.

REFERENCES AND NOTES

1. C. M. Niswender, P. J. Conn, Metabotropic glutamate receptors: Physiology, pharmacology, and disease. *Annu. Rev. Pharmacol. Toxicol.* **50**, 295–322 (2010).

2. R. Crupi, D. Impellizzeri, S. Cuzzocrea, Role of metabotropic glutamate receptors in neurological disorders. *Front. Mol. Neurosci.* **12**, 20 (2019).

3. F. M. Ribeiro, L. B. Vieira, R. G. W. Pires, R. P. Olmo, S. S. G. Ferguson, Metabotropic glutamate receptors and neurodegenerative diseases. *Pharmacol. Res.* **115**, 179–191 (2017).

4. M. L. Parmentier, T. Galvez, F. Acher, B. Peyre, R. Pellicciari, Y. Grau, J. Bockaert, J. P. Pin, Conservation of the ligand recognition site of metabotropic glutamate receptors during evolution. *Neuropharmacology* **39**, 1119–1131 (2000).

5. H. H. Nickols, P. J. Conn, Development of allosteric modulators of GPCRs for treatment of CNS disorders. *Neurobiol. Dis.* **61**, 55–71 (2014).

6. P. Jeffrey Conn, A. Christopoulos, C. W. Lindsley, Allosteric modulators of GPCRs: A novel approach for the treatment of CNS disorders. *Nat. Rev. Drug Discov.* **8**, 41–54 (2009).

7. A. Ellaithy, J. Gonzalez-Maeso, D. A. Logothetis, J. Levitz, Structural and biophysical mechanisms of class C G protein-coupled receptor function. *Trends Biochem. Sci.* **45**, 1049–1064 (2020).

8. N. Kunishima, Y. Shimada, Y. Tsuji, T. Sato, M. Yamamoto, T. Kumasaka, S. Nakanishi, H. Jingami, K. Morikawa, Structural basis of glutamate recognition by a dimeric metabotropic glutamate receptor. *Nature* **407**, 971–977 (2000).

9. D. Tsuchiya, N. Kunishima, N. Kamiya, H. Jingami, K. Morikawa, *Structural views of the ligand-binding cores of a metabotropic glutamate receptor complexed with an antagonist and both glutamate and Gd3+*. **99**, 2660–2665 (2002).

10. T. Muto, D. Tsuchiya, K. Morikawa, H. Jingami, Structures of the extracellular regions of the group II/III metabotropic glutamate receptors. *Proc. Natl. Acad. Sci. U.S.A.* **104**, 3759–3764 (2007).

11. J. A. Monn, L. Prieto, L. Taboada, J. Hao, M. R. Reinhard, S. S. Henry, C. D. Beadle, L. Walton, T. Man, H. Rudyk, B. Clark, D. Tupper, S. R. Baker, C. Lamas, C. Montero, A. Marcos, J. Blanco, M. Bures, D. K. Clawson, S. Atwell, F. Lu, J. Wang, M. Russell, B. A. Heinz, X. Wang, J. H. Carter, B. G. Getman, J. T. Catlow, S. Swanson, B. G. Johnson, D. B. Shaw, D. L. McKinzie, Synthesis and pharmacological characterization of C4-(Thiotriazolyl)-substituted-2-aminobicyclo[3.1.0]hexane-2,6-dicarboxylates. Identification of (1R,2S,4R,5R,6R)-2-Amino-4-(1H-1,2,4-triazol-3-ylsulfanyl)bicyclo[3.1.0]hexane-2,6-dicarboxylic Acid (LY2812223), a highly potent, functionally selective mGlu2 receptor agonist. *J. Med. Chem.* **58**, 7526–7548 (2015).

12. A. Koehl, H. Hu, D. Feng, B. Sun, Y. Zhang, M. J. Robertson, M. Chu, T. S. Kobilka, T. Laeremans, J. Steyaert, J. Tarrasch, S. Dutta, R. Fonseca, W. I. Weis, J. M. Mathiesen, G. Skiniotis, B. K. Kobilka, Structural insights into the activation of metabotropic glutamate receptors. *Nature* **566**, 79–84 (2019).

13. S. Lin, S. Han, X. Cai, Q. Tan, K. Zhou, D. Wang, X. Wang, J. Du, C. Yi, X. Chu, A. Dai, Y. Zhou, Y. Chen, Y. Zhou, H. Liu, J. Liu, D. Yang, M.-W. Wang, Q. Zhao, B. Wu, Structures of G_i-bound metabotropic glutamate receptors mGlu2 and mGlu4. *Nature* **594**, 583–588 (2021).

14. A. B. Seven, X. Barros-Álvarez, M. de Lapeyrière, M. M. Papasergi-Scott, M. J. Robertson, C. Zhang, R. M. Nwokonko, Y. Gao, J. G. Meyerowitz, J.-P. Rocher, D. Schelshorn, B. K. Kobilka, J. M. Mathiesen, G. Skiniotis, G-protein activation by a metabotropic glutamate receptor. *Nature* **595**, 450–454 (2021).

15. J. Du, D. Wang, H. Fan, C. Xu, L. Tai, S. Lin, S. Han, Q. Tan, X. Wang, T. Xu, H. Zhang, X. Chu, C. Yi, P. Liu, X. Wang, Y. Zhou, J.-P. Pin, P. Rondard, H. Liu, J. Liu, F. Sun, B. Wu, Q. Zhao, Structures of human mGlu2 and mGlu7 homo- and heterodimers. *Nature* **594**, 589–593 (2021).

16. C. Nasrallah, G. Cannone, J. Briot, K. Rottier, A. E. Berizzi, C. Y. Huang, R. B. Quast, F. Hoh, J. L. Banères, F. Malhaire, L. Berto, A. Dumazer, J. Font-Ingles, X. Gómez-Santacana, J. Catena, J. Kniazeff, C. Goudet, A. Llebaria, J. P. Pin, K. R. Vinothkumar, G. Lebon, Agonists and allosteric modulators promote signaling from different metabotropic glutamate receptor 5 conformations. *Cell Rep.* **36**, 109648 (2021).

17. J. Zhang, L. Qu, L. Wu, X. Tang, F. Luo, W. Xu, Y. Xu, Z.-J. Liu, T. Hua, Structural insights into the activation initiation of full-length mGlu1. *Protein Cell* **12**, 662–667 (2021).

18. A.-M. Cao, R. B. Quast, F. Fatemi, P. Rondard, J.-P. Pin, E. Margeat, Allosteric modulators enhance agonist efficacy by increasing the residence time of a GPCR in the active state. *Nat. Commun.* **12**, 5426 (2021).

19. L. Olofsson, S. Felekyan, E. Doumazane, P. Scholler, L. Fabre, J. M. Zwier, P. Rondard, C. A. M. Seidel, J.-P. Pin, E. Margeat, Fine tuning of sub-millisecond conformational dynamics controls metabotropic glutamate receptors agonist efficacy. *Nat. Commun.* **5**, 5206 (2014).

20. R. Vafabakhsh, J. Levitz, E. Y. Isacoff, Conformational dynamics of a class C G-protein-coupled receptor. *Nature* **524**, 497–501 (2015).

21. J. Levitz, C. Habrian, S. Bharill, Z. Fu, R. Vafabakhsh, E. Y. Isacoff, Mechanism of assembly and cooperativity of homomeric and heteromeric metabotropic glutamate receptors. *Neuron* **92**, 143–159 (2016).

22. C. H. Habrian, J. Levitz, V. Vyklicky, Z. Fu, A. Hoagland, I. McCort-Tranchepain, F. Acher, E. Y. Isacoff, Conformational pathway provides unique sensitivity to a synaptic mGluR. *Nat. Commun.* **10**, 5572 (2019).

23. W. B. Asher, P. Geggier, M. D. Holsey, G. T. Gilmore, A. K. Pati, J. Meszaros, D. S. Terry, S. Mathiasen, M. J. Kaliszewski, M. D. McCauley, A. Govindaraju, Z. Zhou, K. G. Harikumar, K. Jaqaman, L. J. Miller, A. W. Smith, S. C. Blanchard, J. A. Javitch, Single-molecule FRET imaging of GPCR dimers in living cells. *Nat. Methods* **18**, 397–405 (2021).

24. B. W.-H. Liauw, H. S. Afsari, R. Vafabakhsh, Conformational rearrangement during activation of a metabotropic glutamate receptor. *Nat. Chem. Biol.* **17**, 291–297 (2021).

25. B. Wey-Hung Liauw, A. Foroutan, M. Schamber, W. Lu, H. S. Afsari, R. Vafabakhsh, Conformational fingerprinting of allosteric modulators in metabotropic glutamate receptor 2. *eLife* **11**, e78982 (2022).

26. V. Hlavackova, U. Zabel, D. Frankova, J. Batz, C. Hoffmann, L. Prezeau, J.-P. Pin, J. Blahos, M. J. Lohse, Sequential inter- and intrasubunit rearrangements during activation of dimeric metabotropic glutamate receptor 1. *Sci. Signal.* **5**, ra59 (2012).

27. E. O. Grushevskyi, T. Kukaj, R. Schmauder, A. Bock, U. Zabel, T. Schwabe, K. Benndorf, M. J. Lohse, Stepwise activation of a class C GPCR begins with millisecond dimer rearrangement. *Proc. Natl. Acad. Sci. U.S.A.* **116**, 10150–10155 (2019).

28. V. A. Gutzeit, J. Thibado, D. S. Stor, Z. Zhou, S. C. Blanchard, O. S. Andersen, J. Levitz, Conformational dynamics between transmembrane domains and allosteric modulation of a metabotropic glutamate receptor. *eLife* **8**, e45116 (2019).

29. R. B. Quast, F. Fatemi, M. Kranendonk, E. Margeat, G. Truan, Accurate determination of human CPR conformational equilibrium by smFRET using dual orthogonal noncanonical amino acid labeling. *Chembiochem* **20**, 659–666 (2019).

30. A. Deiters, P. G. Schultz, In vivo incorporation of an alkyne into proteins in Escherichia coli. *Bioorg. Med. Chem. Lett.* **15**, 1521–1524 (2005).

31. A. Deiters, T. A. Cropp, M. Mukherji, J. W. Chin, J. C. Anderson, P. G. Schultz, Adding amino acids with novel reactivity to the genetic code of Saccharomyces cerevisiae. *J. Am. Chem. Soc.* **125**, 11782–11783 (2003).

32. J. K. Takimoto, K. L. Adams, Z. Xiang, L. Wang, Improving orthogonal tRNA-synthetase recognition for efficient unnatural amino acid incorporation and application in mammalian cells. *Mol. Biosyst.* **5**, 931–934 (2009).

33. K. Sakamoto, A. Hayashi, A. Sakamoto, D. Kiga, H. Nakayama, A. Soma, T. Kobayashi, M. Kitabatake, K. Takio, K. Saito, M. Shirouzu, I. Hirao, S. Yokoyama, Site-specific incorporation of an unnatural amino acid into proteins in mammalian cells. *Nucleic Acids Res.* **30**, 4692–4699 (2002).

34. S. Ye, C. Köhrer, T. Huber, M. Kazmi, P. Sachdev, E. C. Y. Yan, A. Bhagat, U. L. RajBhandary, T. P. Sakmar, Site-specific incorporation of keto amino acids into functional G protein-coupled receptors using unnatural amino acid mutagenesis. *J. Biol. Chem.* **283**, 1525–1533 (2008).

35. B. R. Conklin, Z. Farfel, K. D. Lustig, D. Julius, H. R. Bourne, Substitution of three amino acids switches receptor specificity of Gqα to that of Giα. *Nature* **363**, 274–276 (1993).

36. J. S. Italia, C. Latour, C. J. J. Wrobel, A. Chatterjee, Resurrecting the bacterial tyrosyl-tRNA synthetase/tRNA pair for expanding the genetic code of both *E. coli* and *Eukaryotes Chem. Biol.* **25**, 1304–1312.e5 (2018).

37. V. Hong, S. I. Presolski, C. Ma, M. G. Finn, Analysis and optimization of copper-catalyzed azide-alkyne cycloaddition for bioconjugation. *Angew. Chem. Int. Ed. Engl.* **48**, 9879–9883 (2009).

38. S. I. Presolski, V. P. Hong, M. G. Finn, Copper-catalyzed azide-alkyne click chemistry for bioconjugation. *Curr. Protoc. Chem. Biol.* **3**, 153–162 (2011).

39. C. Uttamapinant, A. Tangpeerachaikul, S. Grecian, S. Clarke, U. Singh, P. Slade, K. R. Gee, A. Y. Ting, Fast, cell-compatible click chemistry with copper-chelating azides for biomolecular labeling. *Angew. Chem. Int. Ed. Engl.* **51**, 5852–5856 (2012).

40. C. Besanceney-Webler, H. Jiang, T. Zheng, L. Feng, D. Soriano Del Amo, W. Wang, L. M. Klivansky, F. L. Marlow, Y. Liu, P. Wu, Increasing the efficacy of bioorthogonal click reactions for bioconjugation: A comparative study. *Angew. Chem. Int. Ed. Engl.* **50**, 8051–8056 (2011).

41. P. Scholler, D. Moreno-Delgado, N. Lecat-Guillet, E. Doumazane, C. Monnier, F. Charrier-Savournin, L. Fabre, C. Chouvet, S. Soldevila, L. Lamarque, G. Donsimoni, T. Roux, J. M. Zwier, E. Trinquet, P. Rondard, J.-P. Pin, HTS-compatible FRET-based conformational sensors clarify membrane receptor activation. *Nat. Chem. Biol.* **13**, 372–380 (2017).

42. E. Doumazane, P. Scholler, L. Fabre, J. M. Zwier, E. Trinquet, J.-P. J.-P. Pin, P. Rondard, Illuminating the activation mechanisms and allosteric properties of metabotropic glutamate receptors. *Proc. Natl. Acad. Sci. U.S.A.* **110**, E1416–E1425 (2013).

43. S. Huang, J. Cao, M. Jiang, G. Labesse, J. Liu, J.-P. Pin, P. Rondard, Interdomain movements in metabotropic glutamate receptor activation. *Proc. Natl. Acad. Sci. U.S.A.* **108**, 15480–15485 (2011).

44. K. Lang, J. W. Chin, Bioorthogonal reactions for labeling proteins. *ACS Chem. Biol.* **9**, 16–20 (2014).

45. D. C. Kennedy, C. S. McKay, M. C. B. Legault, D. C. Danielson, J. A. Blake, A. F. Pegoraro, A. Stolow, Z. Mester, J. P. Pezacki, Cellular consequences of copper complexes used to catalyze bioorthogonal click reactions. *J. Am. Chem. Soc.* **133**, 17993–18001 (2011).

46. F. Y. Carroll, A. Stolle, P. M. Beart, A. Voerste, I. Brabet, F. Mauler, C. Joly, H. Antonicek, J. Bockaert, T. Müller, J. P. Pin, L. Prézeau, BAY36-7620: A potent non-competitive mGlu1 receptor antagonist with inverse agonist activity. *Mol. Pharmacol.* **59**, 965–973 (2001).

47. M.-L. Parmentier, L. Prézeau, J. Bockaert, J.-P. Pin, A model for the functioning of family 3 GPCRs. *Trends Pharmacol. Sci.* **23**, 268–274 (2002).

48. F. Ango, L. Prézeau, T. Muller, J. C. Tu, B. Xiao, P. F. Worley, J. P. Pin, J. Bockaert, L. Fagni, Agonist-independent activation of metabotropic glutamate receptors by the intracellular protein Homer. *Nature* **411**, 962–965 (2001).

49. C. Goudet, F. Gaven, J. Kniazeff, C. Vol, J. Liu, M. Cohen-Gonsaud, F. Acher, L. Prezeau, J. P. Pin, Heptahelical domain of metabotropic glutamate receptor 5 behaves like rhodopsin-like receptors. *Proc. Natl. Acad. Sci. U.S.A.* **101**, 378–383 (2004).

50. E. Doumazane, P. Scholler, J. M. Zwier, E. Trinquet, P. Rondard, J.-P. Pin, A new approach to analyze cell surface protein complexes reveals specific heterodimeric metabotropic glutamate receptors. *FASEB J.* **25**, 66–77 (2011).

51. I. Brabet, M. L. Parmentier, C. De Colle, J. Bockaert, F. Acher, J. P. Pin, Comparative effect of l-CCG-I, DCG-IV and γ-carboxy-l-glutamate on all cloned metabotropic glutamate receptor subtypes. *Neuropharmacology* **37**, 1043–1051 (1998).

52. R. Serfling, C. Lorenz, M. Etzel, G. Schicht, T. Böttke, M. Mörl, I. Coin, NAR breakthrough article designer tRNAs for efficient incorporation of non-canonical amino acids by the pyrrolysine system in mammalian cells. *Nucleic Acids Res.* **46**, 1–10 (2017).

53. J. Haubrich, J. Font, R. B. Quast, A. Goupil-Lamy, P. Scholler, D. Nevoltris, F. Acher, P. Chames, P. Rondard, L. Prézeau, J. P. Pin, A nanobody activating metabotropic glutamate receptor 4 discriminates between homo- And heterodimers. *Proc. Natl. Acad. Sci. U.S.A.* **118**, e2105848118 (2021).

54. O. Faklaris, M. Cottet, A. Falco, B. Villier, M. Laget, J. M. Zwier, E. Trinquet, B. Mouillac, J. P. Pin, T. Durroux, Multicolor time-resolved Förster resonance energy transfer microscopy reveals the impact of GPCR oligomerization on internalization processes. *FASEB J.* **29**, 2235–2246 (2015).

55. L. Olofsson, E. Margeat, Pulsed interleaved excitation fluorescence spectroscopy with a supercontinuum source. *Opt. Express* **21**, 3370–3378 (2013).

56. W. Schrimpf, A. Barth, J. Hendrix, D. C. Lamb, PAM: A framework for integrated analysis of imaging, single-molecule, and ensemble fluorescence data. *Biophys. J.* **114**, 1518–1528 (2018).

57. B. Hellenkamp, S. Schmid, O. Doroshenko, O. Opanasyuk, R. Kühnemuth, S. Rezaei Adariani, B. Ambrose, M. Aznauryan, A. Barth, V. Birkedal, M. E. Bowen, H. Chen, T. Cordes, T. Eilert, C. Fijen, C. Gebhardt, M. Götz, G. Gouridis, E. Gratton, T. Ha, P. Hao, C. A. Hanke, A. Hartmann, J. Hendrix, L. L. Hildebrandt, V. Hirschfeld, J. Hohlbein, B. Hua, C.

G. Hübner, E. Kallis, A. N. Kapanidis, J. Y. Kim, G. Krainer, D. C. Lamb, N. K. Lee, E. A. Lemke, B. Levesque, M. Levitus, J. J. McCann, N. Naredi-Rainer, D. Nettels, T. Ngo, R. Qiu, N. C. Robb, C. Röcker, H. Sanabria, M. Schlierf, T. Schröder, B. Schuler, H. Seidel, L. Streit, J. Thurn, P. Tinnefeld, S. Tyagi, N. Vandenberk, A. M. Vera, K. R. Weninger, B. Wünsch, I. S. Yanez-Orozco, J. Michaelis, C. A. M. Seidel, T. D. Craggs, T. Hugel, Precision and accuracy of single-molecule FRET measurements—A multi-laboratory benchmark study. *Nat. Methods* **15**, 669–676 (2018).

58. J. Xu, T. M. Corneillie, E. G. Moore, G.-L. Law, N. G. Butlin, K. N. Raymond, Octadentate cages of Tb(III) 2-hydroxyisophthalamides: A new standard for luminescent lanthanide labels. *J. Am. Chem. Soc.* **133**, 49, 19900–19910 (2011).

59. C. Uttamapinant, A. Tangpeerachaikul, S. Grecian, S. Clarke, U. Singh, P. Slade, K. R. Gee, A. Y. Ting, Fast, Cell-compatible click chemistry with copper-chelating azides for biomolecular labeling. *Angew. Chem. Int. Ed.* **51**, 5852–5856 (2012).
